# Synthesis and properties of RNA constrained by a 2’‐*O*‐disulfide bridge

**DOI:** 10.1002/open.202300232

**Published:** 2024-01-10

**Authors:** Diallo Traoré, Elisa Biecher, Manon Mallet, Sonia Rouanet, Jean‐Jacques Vasseur, Michael Smietana, Christelle Dupouy

**Affiliations:** ^1^ CNRS ENSCM 1919 route de Mende 34293 Montpellier Cedex 5 France

**Keywords:** constrained nucleic acids, disulfide bridge, oligoribonucleotides, phosphoramidites, 2’-OH modification

## Abstract

We recently reported the properties of RNA hairpins constrained by a dimethylene (DME) disulfide (S−S) linker incorporated between two adjacent nucleosides in the loop and showed that this linker locked the hairpin conformation thus disturbing the duplex/hairpin equilibrium. We have now investigated the influence of the length of the linker and synthesized oligoribonucleotides containing diethylene (DEE) and dipropylene (DPE) S−S bridges. This was achieved via the preparation of building blocks, namely 2′‐*O*‐acetylthioethyl (2′‐*O*‐AcSE) and 2′‐*O*‐acetylthiopropyl (2′‐*O*‐AcSP) uridine phosphoramidites, which were successfully incorporated into RNA sequences. Thermal denaturation analysis revealed that the DEE and DPE disulfide bridges destabilize RNA duplexes but do not disrupt the hairpin conformation. Furthermore, our investigation of the duplex/hairpin equilibrium indicated that sequences modified with DME and DEE S−S linkers predominantly lock the hairpin form, whereas the DPE S−S linker provides flexibility. These findings highlight the potential of S−S linkers to study RNA interactions.

## Graphical abstract

This work describes the synthesis and properties of oligoribonucleotides containing diethylene (DEE) and dipropylene (DPE) disulfide bridge between two adjacent uridines.

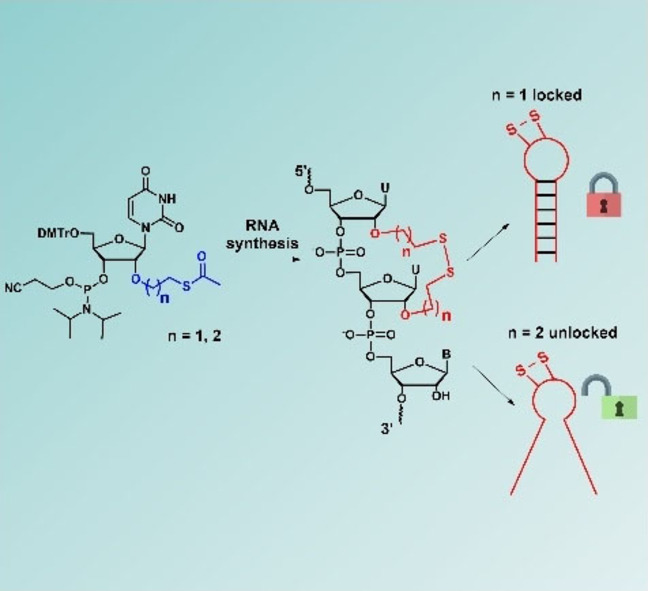

## Introduction

Nucleic acids play vital roles in various biological processes, including regulatory activities, protein interactions, but also in the development of numerous human diseases, such as the recent severe acute respiratory syndrome coronavirus 2 (SARS‐CoV‐2).^[1]^ Recently, modified oligonucleotides, such as antisense oligonucleotides or small interfering RNA (siRNA), are increasingly used as therapeutic agents.^[2]^ Additionally, constrained nucleic acids offer a versatile chemical tool with diverse applications in biology and nanotechnology.^[3]^ Many nucleotides used for therapeutic purposes have been designed to restrict their conformational flexibility, particularly in the sugar moieties.^[4]^ Another strategy relies on constraining nucleic acid structures by imposing conformational restrictions on the sugar‐phosphate backbone. For example, Seeman *et al*. developed a method for synthesizing DNA oligomers through the connection of the 2′ position of two neighboring nucleosides *via* amide bonds.^[5]^ Recently, Seth *et al*. have synthesized constrained nucleic acids relying on connected adjacent backbones with phosphonate esters bridged by hydrocarbon linkers, holding potential for therapeutic applications.^[6]^ Furthermore, other types of constrained modifications have been utilized to regulate the folding of nucleic acid structures.^[7]^ While, the majority of these constrained structures are formed through covalent linkages, limited attention has been given to dynamic systems relying on structural units such as disulfide bounds (S−S). Among the many dynamic chemical bonds, S−S linkages emerged as a valuable tool in nucleic acids for bioconjugation and therapeutic applications.^[8]^

**Figure 1:**
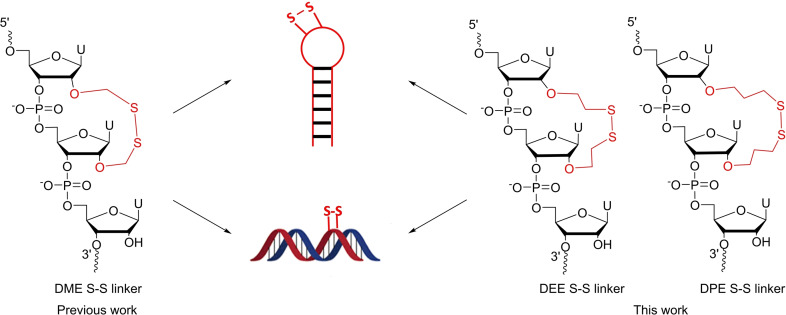
Incorporation of DME, DEE and DPE S−S linker into duplexes and hairpins.

In this context, we previously described the synthesis of 2’‐*O*‐acetyldithiomethyl uridine phosphoramidite which induced the formation of a dimethylene disulfide (DME) bridge when two residues were introduced into oligoribonucleotides side by side (Figure 1).^[9]^ We showed that when this bridge was incorporated into RNA duplexes, a strong thermal destabilization was observed. In contrast, when the disulfide bridge was introduced in the center of the loop of RNA hairpins, thermal stability was increased up to Δ*T_m_* +2.7 °C compared to their non‐modified counterparts and the hairpin was remained 70 % intact against 3′ exonuclease activity after 24 hours.

Furthermore, the influence of the S−S bridge was studied in RNA hairpin / duplex equilibrium of the DIS sequence involved in the dimerization initiation site of HIV‐1.^[9b]^ We have shown that the S−S bridge disturbed the duplex / hairpin equilibrium by locking the hairpin form while the addition of a reducing agent, cleaved the disulfide bridge and restored the extended duplex.

These results prompted us to examine the influence of the length of the alkyl linker on the folding behavior of RNA duplexes and hairpins. For this purpose, we present here the synthesis of oligoribonucleotides (ORNs) incorporating diethylene (DEE) and dipropylene (DPE) disulfide bridges as well as their influence on the thermal and enzymatic stabilities compared to analogous systems containing the DME linker (Figure 1).

## Results and Discussion

### Synthesis of oligoribonucleotides modified by a 2’S−S bridge

**Scheme 1:**
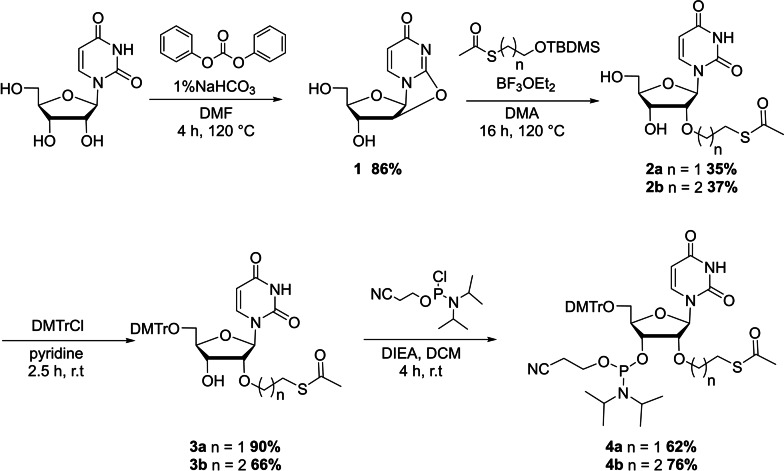
Synthesis of 2’‐*O*‐(2‐acetylthioethyl) (2’‐*O*‐AcSE) **4 a** and 2’‐*O*‐(2‐acetylthiopropyl) (2’‐*O*‐AcSP) **4 b** uridine phosphoramidites.

**Scheme 2:**
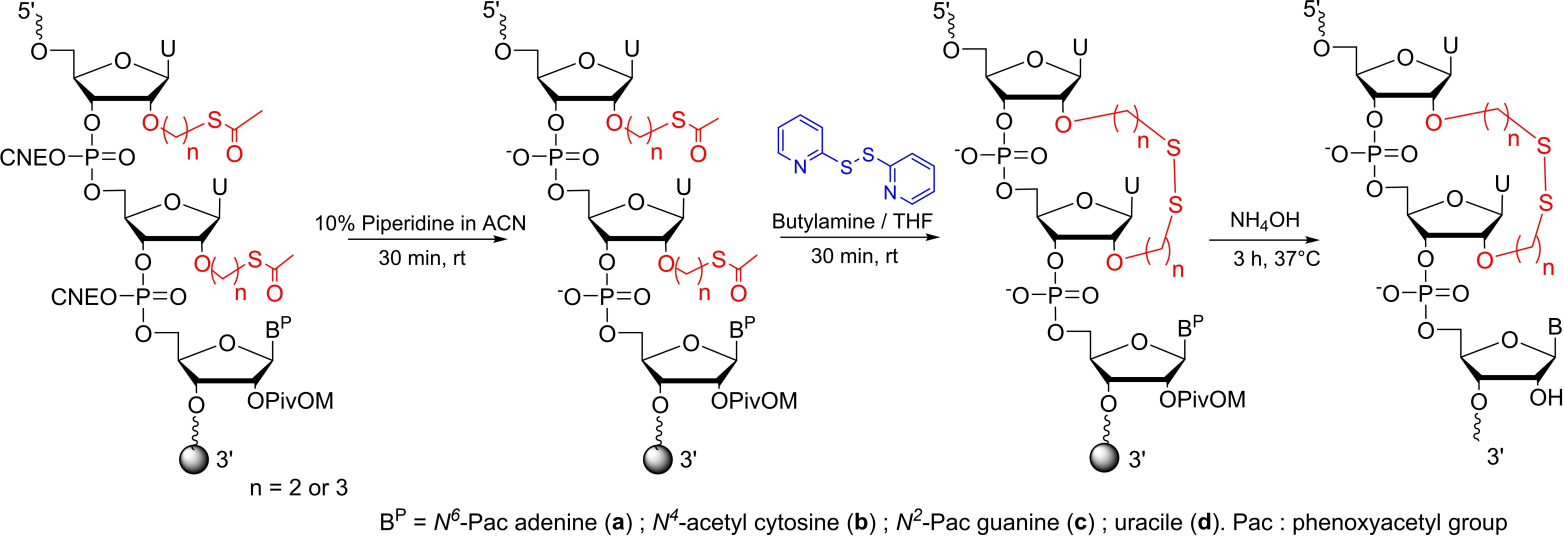
Synthesis of modified oligonucleotides containing DEE and DPE disulfide linker.

The formation of the DEE and DPE disulfide bridges between two adjacent nucleosides is adapted from a strategy previously described by us.^[9b]^ The modified ORNs required the preparation of 2’‐*O*‐(2‐acetylthioethyl) (2’‐*O*‐AcSE) and 2’‐*O*‐(2‐acetylthiopropyl) (2’‐*O*‐AcSP) uridine phosphoramidites. Using uridine as starting material, the synthesis was initiated with the formation of 2,2’‐anhydrouridine **1** in presence of diphenyl carbonate and a catalytic amount of sodium bicarbonate.^[10]^ Treatment of **1** with 2‐S‐acetyl‐*tert*‐butyl‐dimethylsilyl ethanol or 2‐S‐acetyl‐*tert*‐butyl‐dimethylsilyl propanol in presence of boron trifluoride diethyl etherate generated the corresponding compounds **2 a** and **2 b** respectively.^[11]^ The 5′‐hydroxyl function of these compounds was then protected with a dimethoxytrityl group and a subsequent phosphitylation allow us to obtain the corresponding phosphoramidites **4 a** and **4 b** with an overall yield of 17 % and 13 % respectively (Scheme 1). With these new phosphoramidites building blocks in hand we then synthesized three singles stranded ORNs containing the DME (**ON1**, ^5’^GCCUUC**U_1_U_1_** AUGAUU^3’^), DEE (**ON2**, ^5’^GCCUUC**U_2_U_2_** AUGAUU^3’^) and DPE (**ON3**, ^5’^GCCUUC**U_3_U_3_** AUGAUU^3’^) S−S linker in the middle of the sequence (Scheme 2).

To study the influence of these modifications on hairpins, we also synthesized partially self‐complementary RNA with the sequence **H2**–**H7** (Table 1) containing the constrained dinucleotides with the S−S linker in the loop part.^[9b]^ The ORNs were prepared according to the RNA synthesis method previously described by us.^[12]^

**Table 1:** Thermal denaturation studies of duplexes and hairpins.

ORN	Duplexes and Hairpins^[a]^	T_m_ (°C)^[b]^	ΔT_m_ (°C)^[b]^
D1	^5’^GCCUUCUUAUGAUU^3’^ ^3’^CGGAAGAAUACUAA^5’^	58.2	/
D2	^5’^GCCUUC**U_1_U_1_** AUGAUU^3’^ ^3’^CGGAAGAAUACUAA^5’^	48.0	−10.1
D3	^5’^GCCUUC**U_2_U_2_** AUGAUU^3’^ ^3’^CGGAAGAAUACUAA^5’^	38.6	−19.6
D4	^5’^GCCUUC**U_3_U_3_** AUGAUU^3’^ ^3’^CGGAAGAAUACUAA^5’^	47.3	−10.9
D5	^5’^GCCUUCUUAUGAUU^3’^ ^3’^d(CGGAAGAATACTAA)^5’^	38.7	/
D6	^5’^GCCUUC**U_1_U_1_** AUGAUU^3’^ ^3’^d(CGGAAGAATACTAA)^5’^	23.7	−15.0
D7	^5’^GCCUUC**U_2_U_2_** AUGAUU^3’^ ^3’^d(CGGAAGAATACTAA)^5’^	15.3	−23.4
D8	^5’^GCCUUC**U_3_U_3_** AUGAUU^3’^ ^3’^d(CGGAAGAATACTAA)^5’^	25.7	−13.0
H1	^5’^AUCCAGUUUUUGGAU^3’^	61.2	
H2	^5’^AUCCAG**U_1_U_1_** UUUGGAU^3’^	61.2	0
H3	^5’^AUCCAGU**U_1_U_1_** UUGGAU^3’^	61.2	0
H4	^5’^AUCCAG**U_2_U_2_** UUUGGAU^3’^	60.0	−1.2
H5	^5’^AUCCAGU**U_2_U_2_** UUGGAU^3’^	60.3	−0.9
H6	^5’^AUCCAG**U_3_U_3_** UUUGGAU^3’^	58.5	−2.7
H7	^5’^AUCCAGU**U_3_U_3_** UUGGAU^3’^	60.0	−1.2

[a] **U_1_U_1_** =2’,2’ DME S−S bridge; **U_2_U_2_** =2’,2’ DEE S−S bridge; **U_3_U_3_** =2’,2’ DPE S−S bridge. [b] *T_m_* values obtained from UV melting curves at 260 nm with 1.5 μM strand concentration in 10 mM sodium cacodylate, 100 mM NaCl pH 7. Data are averages of two denaturation/hybridization cycles. Estimated errors in *T_m_*: ±0.5 °C.

The ORNs containing the DME linker, were previously obtained on solid support by treatment with 2,2’‐dithiopyridine in presence of butylamine, which removed concomitantly the cyanoethyl protecting groups.^[9b]^ We noticed however, that under the exact same conditions an additional peak corresponding to [M+108]^+^ was observed in MALDI‐TOF MS analysis for **ON2** and **ON3**.

This side product was attributed to a double Michael addition of acrylonitrile, to the deprotected thiol functions. A two‐step deprotection process was thus developed to avoid the formation of this side product involving first, the treatment of the CPG beads with a 10 % piperidine solution in acetonitrile during 30 min to cleave the cyanoethyl protections, subsequent washing with acetonitrile to remove acrylonitrile and then the addition of 20 equivalents of 2,2’‐dithiopyridine in presence of butylamine. This two‐steps procedure allowed the exclusive formation of the S−S bridge.

At the end of RNA elongation on solid support, a standard ammonia treatment was applied to remove all remaining protecting groups and to release the ORNs from the solid support. Crudes were purified by IEX‐HPLC and characterized by MALDI‐TOF Mass spectrometry (Table S1).

### Thermal denaturation and CD studies of duplexes

The thermal stabilities of the duplexes formed with **ON1**, **ON2** and **ON3** and their complementary DNA and RNA strands were determined by UV melting experiments at 260 nm. As indicated in Table 1, whatever the length of the S−S linker, the modification induced a strong destabilization on RNA/RNA and RNA/DNA duplexes compared to the corresponding unmodified one ranging from Δ*T_m_* =−10.1 to −23.4 °C .

Among the three evaluated bridges, the DEE S−S linker induced the most important destabilization both in the presence of a complementary RNA (**D3** with Δ*T_m_* =−19.6 °C, Table 1) and DNA (**D7** with Δ*T_m_* =−23.4 °C, Table 1) strands.

Circular dichroism (CD) spectra of unmodified **D1** and modified **D2**, **D3** and **D4** RNA/RNA duplexes showed the typical A‐form double helical geometry with a negative band at 210 nm and a positive band at approximately 265 nm demonstrating that the A‐form structure is conserved (Figure S21). For the unmodified DNA/RNA duplex **D5**, the CD spectrum showed a characteristic mixed A and B pattern with a positive band around 270 nm (Figure S22). The CD intensities of modified RNA/DNA duplexes **D6**–**D8** decreased suggesting that the disulfide bridge might affect the base pair stacking interaction.

### Studies of duplex /hairpin equilibrium

We previously studied the duplex / hairpin equilibrium with HIV‐DIS sequence and reported that the DME S−S linker locked the hairpin form when it was introduced into the middle of a loop of the DIS sequence.^[9b]^ In order to evaluate the influence of the DEE and DPE S−S linker on the hairpin/duplex interconversion, we employed the 14‐mer sequence **H1** as a model (Table 1).^[13]^ In the absence of a complementary strand, the sequence adopts a hairpin structure composed of five nucleotides in the loop part. In contrast, a duplex is formed in presence of the complementary strand. We thus designed modified sequences in which the DME, DEE and DPE S−S linker were introduced between two uridines adjacent of the loop part (U7 and U8 for **H2**, **H4**, **H6** and U8 and U9 for **H3**, **H5**, **H7**, Table 1).

Thermal denaturation studies of hairpins **H1** and **H2** confirmed that the DME S−S bridge do not induce a destabilization when incorporated in the center of the loop.^[9b]^ Compared to the unmodified hairpin, the DEE and DPE S−S linkers induce a slight destabilization ranging from Δ*T_m_* =−0.9 °C for **H4** to Δ*T_m_* =−2.9 °C for **H5** demonstrating that regardless of the length of the linker, the S−S bridge is well‐tolerated when positioned in the center of the hairpin loop CD spectra of unmodified hairpins **H1** and modified **H2**–**H7** hairpins exhibited the characteristic curve of A form indicating that the disulfide bridges did not disturb the hairpin structure (Figure S23). It is noteworthy that the CD intensity of **H7** was decreased compared to the unmodified one suggesting that the DPE linker might have an impact on the base pairing interactions.

**Figure 2:**
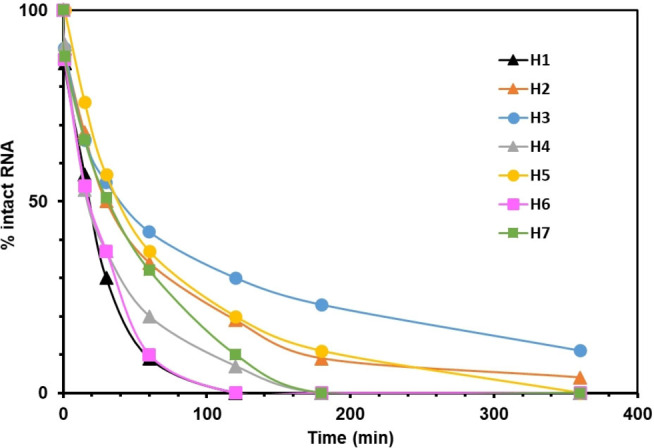
Enzymatic stability of unmodified hairpin **H1** and modified hairpins **H2‐H7** containing DME, DEE and DPE disulfide linker against SVPDE.

A 3’‐exonuclease (snake venom phosphodiesterase (SVPDE)) was used in order to evaluate the influence of these modifications against enzymatic digestion. The digestion was monitored by IEX‐HPLC and the percentage of intact ON was determined at different time (Figure 2). The half‐lives of hairpins **H4** and **H6**, with the linker positioned between U7 and U8, were found to be identical to the unmodified hairpin **H1** (t_1/2_=20 min). In contrast, hairpins containing the S−S linker between U8 and U9 exhibited the highest resistance to SVPDE, with a half‐life of 40 min for **H3** and **H5**. Interestingly, after 120 min, the HPLC chromatogram for **H3** displayed multiple peaks, while hairpin **H1** was completely degraded. MALDI‐TOF mass analysis revealed that the major peaks corresponded to 14‐mer and 10‐mer fragments (Figure S24).

For hairpins **H6** and **H7**, which contained the DPE S−S linker, the ONs were almost completely degraded after 120 min. These findings suggest that the position of the DME and DEE S−S bridges can impact the activity of the 3′‐exonuclease. The nature of the linker also influences the stability of the hairpins. Indeed, the incorporation of a longer linker decreases the enzymatic stability.

**Figure 3:**
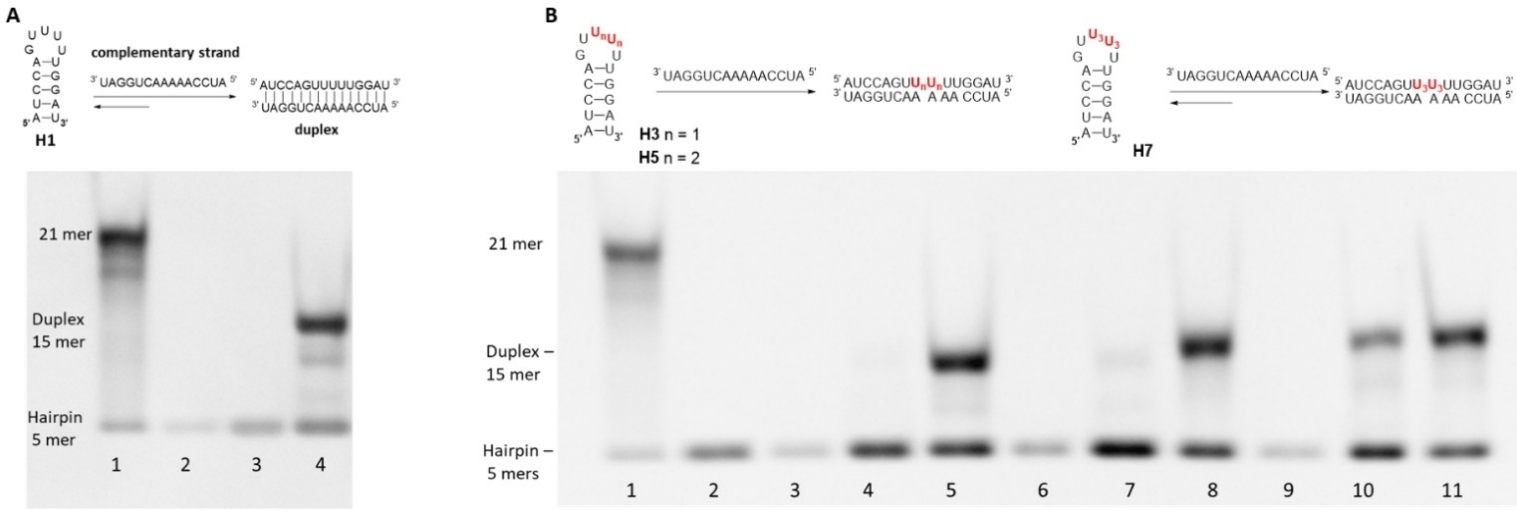
Gel electrophoresis to study hairpin‐duplex equilibrium. All samples were denatured by heating at 90 °C for 3–5 min followed by cooling to room temperature overnight; the samples were then stored at 4 °C for 3 h and diluted with formamide (20 μL). Migration in TBE buffer at 4 °C (**A**) gel electrophoresis with hairpin H1 and complementary strand 5’‐AUCCAAAAACUGGAU‐3’ (lane 1 reference: 21mer RNA duplex, lane 2: **H1**, lane 3: complementary strand, lane 4: **H1**+complementary strand). (**B)** gel electrophoresis with sequences **H3**, **H5** and **H7** and complementary strand ^5’−^AUCCAAAAACUGGAU (lane 1 reference: 21mer RNA duplex, lane 2: **H3**, lane 3: complementary strand, lane 4: **H3**+complementary strand, lane 5: **H3**+complementary strand +TCEP, lane 6: **H5**, lane 7: **H5**+complementary strand, lane 8: **H5**+complementary strand+TCEP, lane 9: **H7**, lane 10: **H7**+complementary strand, lane 11: **H7**+complementary strand+TCEP)Sequences for the study of Hairpin/duplex equilibrium. **U_1_U_1_** =2’,2’ DME S−S bridge; **U_2_U_2_** =2’,2’ DEE S−S bridge; **U_3_U_3_** =2’,2’ DPE S−S bridge.

To further study the impact of the three modified S−S bridges on the equilibrium between duplex and hairpin structures non‐denaturing gel electrophoresis (15 % polyacrylamide) were performed at 4 °C (Figure 3) using hairpins **H1**, **H3**, **H5** and **H7** along with their complementary strands. In the case of the unmodified **H1** sequence, the gel analysis revealed the exclusive presence of the hairpin form (Figure 3A, lane 2 and 3). However, upon introducing the complementary strand, two distinct bands were observed. The more prominent band indicated the presence of the duplex form (around 70 %, determined by Image J Software), suggesting a preference for the duplex over the hairpin conformation. In contrast, when examining **H3**, a band corresponding to the hairpin structure was majorly observed in the absence or presence of the complementary strand (Figure 3B, lane 4). However, upon addition of tris(2‐carboxyethyl)phosphine (TCEP), a retarded band appeared, indicating the formation of the duplex structure (around 62 %) resulting from the reduction of the S−S bond (Figure 3B, lane 5). These findings align with our previous reports, further validating our earlier observations.^[9b]^ Similar observations were made with **H5**. Indeed, the DEE linker effectively stabilized the hairpin form, while the addition of TCEP resulted in the formation of 66 % of the duplex structure (Figure 3B, lane 8). In contrast, when examining the sequence with the DPE S−S linker (**H7**) the addition of its complementary strand, revealed the presence of two distinct bands, corresponding to the hairpin and duplex forms respectively (Figure 3B, lane 10). The analysis indicated that approximately 40 % of the structure existed in the duplex form, implying a certain degree of flexibility in the overall structure. With the addition of TCEP, the duplex structure becomes dominant, accounting for approximately 64 % (Figure 3B, lane 11). This result implies that the incorporation of the DPE modification did not lock the ORN into the hairpin conformation, due to the increased flexibility compared to shorter linkers.

It is well known that RNA exist in a wide variety of structural conformations.^[14]^ To gain some insight about the influence of the S−S link the conformations of 2’,2’ DME S−S, 2’,2’ DEE S−S and 2’,2’ DPE S−S dinucleotides were investigated though energy minimization using Avogadro software and compared to the unmodified UU. In particular we evaluated the O3′‐P_i+1_‐O5′_i+1_ phosphodiester bound defined by torsion angles α and ζ as well as the C1′‐C1′_i+1_ distance.

The unmodified UU dimer was found as expected with stacked bases while the 2’,2’ DME S−S, 2’,2’ DEE S−S and 2’,2’ DPE S−S dinucleotides were characterized by two widely extended bases implying limited or no stacking at all. The results, detailed in the Supplementary information (Figure S25 and Table S2), confirm that the S−S link is not well‐suited for duplex formation but can be better tolerated in unpaired regions, such as the loop part of hairpin structures.

## Conclusions

In this study, we accomplished the successful synthesis in four steps of 2′‐*O*‐acetylthioethyl (2′‐*O‐*AcSE) and 2′‐*O*‐acetylthiopropyl (2′‐*O*‐AcSP) uridine phosphoramidites, starting from uridine. These modifications were efficiently incorporated into ORNs, allowing the formation of DEE and DPE disulfide bridges between adjacent nucleosides through a thiol disulfide exchange reaction on a solid support. The effects of these modifications were thoroughly investigated and compared to sequences containing the previously reported DME S−S linker. Upon introducing these modifications into RNA/RNA and RNA/DNA duplexes, a notable destabilization effect was observed. However, when the modified sequences were incorporated in the middle of a loop part of a hairpin, the DME, DEE and DPE S−S linkers were well tolerated. Furthermore, the presence of the DME and DEE S−S linkers slowed down the enzymatic activity of the 3’‐exonuclease.

Additionally, the investigation of the equilibrium between hairpin and duplex structures demonstrated that the DME and DEE S−S linkers effectively locked the conformation of the ORNs in the hairpin form. In contrast, the DPE S−S linker exhibited greater flexibility, allowing for a more dynamic equilibrium between hairpin and duplex forms. This variation of length in disulfide bridges presents an exciting opportunity to use them as tools for *in vitro* studying RNA folding, mismatch detection or interaction of RNA with proteins.

## Experimental section

Pyridine and DIEA were distilled over calcium hydride. All reactions were performed in anhydrous conditions under argon. All other solvents and reagents were purchased from commercial suppliers and were used without purification. NMR experiments were accomplished on Bruker DRX 400 spectrometer at 20 °C. HRMS analyses were obtained with electrospray ionization (ESI) in positive or negative mode on a Q‐TOF Micromass spectrometer. ORNs were synthesized using an automated DNA synthesizer (Applied Biosystems 394). Crude ONs were analyzed and purified by IEX‐HPLC on a Dionex DNAPac® PA100, 4×250 mm column for analysis or a 9×250 column for semi‐preparative purpose. The following HPLC solvent system was used: 5 % CH_3_CN in 25 mM Tris‐HCl pH 8 (buffer A), 5 % CH_3_CN containing 400 mM NaClO_4_ in 25 mM Tris‐HCl pH 8 (buffer B). Flow rates were 1.0 mL/min for analysis or 4 mL/min for semi‐preparative purpose; UV detection was performed at 260 nm. MALDI‐TOF mass spectra were recorded using a Shimadzu AXIMA Assurance spectrometer equipped with an N_2_ laser (337 nm) (Shimadzu, Japan) using 2,1,1‐trihydroxyacetophenone as a saturated solution in a mixture of acetonitrile/0.1 M ammonium citrate solution (1 : 1, v/v) for the matrix. Analytical samples were mixed with the matrix in the 1 : 5 (v/v) ratio, crystallized on a 384‐well stainless‐steel plate and analyzed. UV quantitation of RNAs was performed using a Varian Cary 300 Bio UV/Visible spectrometer by measuring absorbance at 260 nm. Lyophilized ORNs were stored at −20 °C for several months without any degradation.

**2’‐*O*‐acetylthioethyl uridine (2 a)**. 2,2’‐Anhydrouridine **1** (1 equiv.) was dried over P_2_O_5_ for 5 hours and was coevapored three times with anhydrous pyridine, three time with anhydrous toluene and dissolved in DMA (2 mL/ mmol) under argon. 2‐S‐acetyl‐*tert*‐butyl‐dimethylsilyl ethanol or 2‐S‐acetyl‐*tert*‐butyl‐dimethylsilyl propanol (5 equiv.) and boron trifluoride diethyl etherate (2.5 equiv.) were added and the mixture was stirred at 120 °C for 16 h. Ethanol was added and the solution was concentrated under vacuum. The crude material was purified by silica gel column chromatography with a step gradient of methanol in dichloromethane (0–5 %). Compound **2 a** was obtained as a white solid (35 % yield). ^1^H NMR (400 MHz, DMSO d_6_): *δ*: 11.34 (d, *J*=1.6 Hz, 1H, NH), 7.92 (d, *J*=8.1 Hz, 1H, H_6_), 5.81 (d, *J*=4.7 Hz, 1H, H_1’_), 5.64 (dd, *J*=8.1, 2.1 Hz, 1H, H_5_), 5.13 (t, *J*=5.1 Hz, 1H, 5’‐OH), 5.12 (d, *J*=6.0 Hz, 1H, 3’‐OH), 4.07 (td, *J*=5.5, 5.2 Hz, 1H, H_3’_), 3.92 (t, *J*=4.8 Hz, 1H, H_2’_), 3.85–3.82 (m, 1H, H_4’_), 3.71 (td, *J*=10.2, 6.5 Hz, 1H, *H*‐CH), 3.65 (ddd, *J*=12.2, 5.2, 3.1 Hz, 1H, H_5’_), 3.53–3.60 (m, 2H, H_5’_ and *H*‐CH), 3.09–2.96 (m, 2H, CH_2_), 2.31 (s, 3H, CH_3_); ^13^C NMR (100 MHz, DMSO d_6_): *δ*: 195.1, 163.2, 150.5, 140.4, 101.8, 86.2, 84.8, 81.4, 68.3, 68.2, 60.3, 30, 28.3. HRMS (ESI^+^) m/z calcd for C_13_H_19_N_2_O_7_S_1_ [M+H]^+^ 347.0907, Found 347.0919.

**2’‐*O*‐acetylthiopropyl uridine (2 b)**. Using the same procedure as for the synthesis of **2 a**, compound **2 b** was obtained as a white solid (37 % yield). ^1^H NMR (400 MHz, DMSO d_6_) *δ*: 11.32 (s, 1H, NH), 7.92 (d, *J*=8.1 Hz, 1H, H_6_), 5.83 (d, *J*=4.9 Hz, 1H, H_1’_), 5.64 (d, *J*=8.0 Hz, 1H, H_5_), 5.14 (t, *J*=5.0 Hz, 1H, 5’‐OH), 5.09 (d, *J*=5.9 Hz, 1H, 3’‐OH), 4.12–4.07 (m, 1H, H_3’_), 3.87–3.84 (m, 2H, H_2’_ and H_4’_), 3.67‐3.47 (m, 4H, H_5’_ and CH_2_) 2.86 (t, *J*=7.2 Hz, 2H, CH_2_), 2.30 (s, 3H, CH_3_), 1.77–1.72 (m, 2H, CH_2_); ^13^C NMR (100 MHz, DMSO‐d_6_ **)** *δ*: 195.8, 163.6, 151.0, 140.8, 102.2, 86.6, 85.5, 81.7, 68.8, 68.7, 60.9, 31.0, 29.6, 25.7; HRMS (ESI^+^) m/z calcd for C_14_H_21_N_2_O_7_S_1_ [M+H]^+^ 361.1064, Found 361.1068.

**2’‐*O*‐acetylthioethyl‐5’‐*O*‐(4,4’‐dimethoxytrityl) uridine (3 a)**. Compound **2 a** (1.00 g, 2.89 mmol, 1 equiv.) was coevapored three times with anhydrous pyridine and diluted in anhydrous pyridine under argon atmosphere. To the solution was added 4,4’‐dimethoxytrityl chloride (1.22 g, 3.61 mmol, 1.25 equiv.) in 7 times over 1 h, and the solution was stirred for 2.5 h. The solution was then diluted in ethyl acetate and poured into saturated NaHCO_3_ solution. The aqueous layer was extracted three times with ethyl acetate. The combined organic layers were washed with brine, dried over anhydrous Na_2_SO_4_ and concentrated under vacuum. The crude material was purified by silica gel column chromatography with a step gradient of methanol in dichloromethane (0–2 %, containing 1 % pyridine). Compound **3 a** was obtained as a white solid (90 % yield).^1^H NMR (400 MHz, CDCl_3_) *δ*: 8.74 (bs, 1H, NH), 8.03 (d, *J*=8.2 Hz, 1H, H_6_), 7.41–7.13 (m, 9H, Ar−H), 6.84 (d, *J*=8.9 Hz, 4H, Ar−H), 5.91 (d, *J*=0.8 Hz, 1H, H_1’_), 5.27 (d, *J*=8.2 Hz, 1H, H_5_), 4.46 (td, *J*=8.7, 5.3 Hz, 1H, H_3’_), 4.10 (td, *J*=10.4, 5.9 Hz, 1H, *H*‐CH), 3.99 (td, *J*=8.2, 2.2 Hz, 1H, H_4’_), 3.93 (d, *J*=5.2 Hz, 1H, H_2’_), 3.83–3.77 (m, 7H, OCH_3_ and *H*‐CH), 3.57 (ddAB, *J*=2.2, 11.2 Hz, 1H, H_5’_), 3.53 (ddAB, *J*=2.6, 11.2 Hz, 1H, H_5’_), 3.21 (ddd, *J*=14.1, 6.5, 5.3 Hz, 1H, *H*‐CH), 3.11 (ddd, *J*=14.1, 6.8, 5.4 Hz, 1H, *H*‐CH), 2.65 (d, *J*=9.5 Hz, 1H, 3’‐OH), 2.35 (s, 3H, CH_3_); ^13^C NMR (100 MHz, CDCl_3_) *δ*: 195.4, 163.4, 158.9, 158.8, 144.5, 135.4, 135.2, 150.2, 140.0, 130.3, 130.2, 129.2, 128.4, 128.3, 128.1, 127.3, 125.4, 113.4, 102.2, 87.7, 83.4, 82.9, 69.6, 68.5, 61.2, 55.4, 30.7, 29.1; HRMS (ESI^+^) m/z calcd for C_34_H_36_N_2_O_9_S_1_ [M+H]^+^ 649.2220, Found 649.2223.

**2’‐*O*‐acetylthiopropyl‐5’‐*O*‐(4,4’‐dimethoxytrityl) uridine (3 b)**. Using the same procedure as for the synthesis of **3 a**, compound **3 b** was obtained as a white solid (66 % yield). ^1^H NMR (400 MHz, CDCl_3_) *δ*: 8.53 (bs, 1H, NH), 8.04 (d, *J*=8.0 Hz, 1H, H_6_), 7.39–7.15 (m, 9H, Ar−H), 6.86–6.83 (m, 4H, Ar−H), 5.91 (d, *J*=0.8 Hz, 1H, H_1’_), 5.25 (d, *J*=8.0 Hz, 1H, H_5_), 4.48 (td, *J*=8.4, 5.2 Hz, 1H, H_3’_), 4.05–4.01 (m, 2H, H_4’_ and *H*‐CH), 3.87(dd, *J*=5.2, 0.8 Hz, H_2’_), 3.79 (s, 6H, OCH_3_), 3.69–3.64 (m, 1H, *H*‐CH), 3.59‐3.52 (m, 2H, H_5’_), 3.09–3.03 (m, 1H, *H*‐CH), 2.96–2.93 (m, 1H, *H*‐CH), 2.88 (d, *J*=9.2 Hz, 1H, 3’‐OH), 2.33 (s, 3H, CH_3_), 1.92 (quint., J=6.4 Hz, 2H, CH_2_); ^13^C NMR (100 MHz, CDCl_3_) *δ*: 196.1, 163.4, 158.8, 158.7, 150.1, 144.4, 140.1, 135.3, 135.1, 130.2, 130.1, 129.0, 128.2, 128.1, 128.0, 127.2, 125.3, 113.3, 102.0, 87.1, 83.3, 82.6, 68.9, 68.4, 61.1, 55.3, 30.7, 29.6, 25.6; HRMS (ESI^+^) m/z calcd for C_35_H_39_N_2_O_9_S [M+H]^+^ 663.2332, Found 663.2371.

**2’‐*O*‐acetylthioethyl‐3’‐*O*‐(2‐cyanoethyl‐*N,N‐*diisopropylphosphoramidite)‐5’‐*O*‐(4,4’‐dimethoxytrityl) uridine (4 a)**. To a solution of **3 a** (1.17 g, 1.80 mmol, 1 equiv.) in anhydrous CH_2_Cl_2_ (18 mL) was added dropwise N,N‐diisoproylethylamine (0.35 mL, 1.98 mmol, 1.1 equiv) and 2‐cyanoethyl N,N‐diisopropylchlorophosphoramidite (0.48 mL, 2.16 mmol, 1.2 equiv.) in anhydrous CH_2_Cl_2_ (3 mL). The mixture was stirred for 2.5 h at room temperature under argon. After reaction completion, ethyl acetate previously washed with a saturated aqueous NaHCO_3_ solution was added and the reaction mixture was poured into a saturated NaCl / NaHCO_3_ solution (1/1 v/v). The aqueous layer was extracted with ethyl acetate and the organic layers were dried over Na_2_SO_4_. The solvent was concentrated under reduced pressure? The crude material was purified by silica gel column chromatography with an isocratic elution of CH_2_Cl_2_ and ethyl acetate containing 1 % pyridine. The desired compound **4 a** was obtained as a white foam (62 % yield). ^1^H NMR (400 MHz, CD_3_CN) *δ*:8.97 (s, 1H, NH), 7.81 (d, *J*=8.2 Hz, 1H, H_6_ dias 1), 7.73 (d, *J*=8.2 Hz, 1H, H_6_ dias 2), 7.51–6.83 (m, 13H, Ar−H, dias 1,2), 5.84–5.80 (m, 1H, H_1’_, dias 1,2), 5.21–5.19 (m, 1H, H_5’_, dias 1,2), 4.49 (ddd, *J*=9.6, 7.0, 5.0 Hz, 1H, H_3’_, dias 1), 4.42 (ddd, *J*=9.9, 6.7, 4.9 Hz, 1H, H_3’_, dias 2), 4.17 – 4.08 (m, 1H, H_2_’, dias1,2), 4.08–4.06 (m, 1H, H4’,dias1,2), 3.91–3.82‐3.78 (m, 2H, CH_2_ dias1,2), 3.77 (s, 6H, OCH_3_ dias 1,2), 3.69–3.52 (m, 4H, CH(iPr)_2_ dias1,2), 3.47–3.40 (m, 2H, H_5’_ dias1,2), 3.14–3.02 (m, 2H, CH_2_ dias1,2), 2.68 (t, *J*=5.9 Hz, 2H, CH_2_ dias1,2), 2.58–2.47 (m, 2H, CH_2_ dias1,2), 2.30 (s, 3H, CH_3_ dias1,2), 1.17 (m, 12H, (iPr‐CH_3_)_2_ dias1,2); ^31^P NMR (121 MHz, CD_3_CN*) δ*:149.7, 149.0; HRMS (ESI^+^) m/z calculated for C_43_H_54_N_4_O_10_PS [M+H]^+^ 849.3254, found 849.3293.

**2’‐*O*‐acetylthiopropyl‐3’‐*O*‐(2‐cyanoethyl‐*N,N‐*diisopropylphosphoramidite)‐5’‐*O*‐(4,4’‐dimethoxytrityl) uridine (4 b)**. Using the same procedure as for the synthesis of **4 a**, compound **4 b** was obtained as a white solid (76 % yield).^1^H NMR (400 MHz, CD_3_CN) *δ*: 8.99 (s, 1H, NH), 8.49–8.48 (m, 2H, H_6_, dias1,2), 7.75–7.63 (m, 2H, Ar−H, dias 1,2), 7.39–7.16 (m, 10H, Ar−H, dias 1,2), 6.82–6.78 (m, 4H, Ar−H, dias 1,2), 5.77 (d, *J*=3.6 Hz, 1H, H_1’_, dias 1), 5.75 (d, *J*=2.8 Hz, 1H, H_1’_, dias,2), 5.14–5.11 (m, 1H, H_5_, dias 1,2), 4.41 (ddd, *J*=10.0, 6.7, 4.9 Hz, 1H, H_3’_ dias 1), 4.34 (ddd, *J*=10.0, 6.7, 4.9 Hz, 1H, H_3’_ dias 2), 4.10‐4.04 (m, 1H, H_2’_ dias 1,2), 3.99–3.92(m, 1H, H_4’_ dias 1,2), 3.80–3.74 (m, 2H, CH_2_ dias1,2), 3.71–3.59 (m, 4H, CH(iPr)_2_ dias1,2), 3.69 and 3.68 (s, 6H, OCH_3_ dias 1,2), 3.57–3.47 (m, 2H, CH_2_ dias1,2), 3.37–3.27 (m, 2H, H_5’_ dias1,2), 2.88–2.82 (m, 2H, CH_2_ dias1,2), 2.60 (t, *J*=6.0 Hz, 2H, CH_2_ dias1), 2.44 (t, *J*=6.0 Hz, 2H, CH_2_ dias1), 1.78–1.70 (m, 2H, CH_2_ dias1,2), 1.01–0.96 (m, 12H, iPr‐CH_3_)_2_ dias1,2); ^31^P NMR (121 MHz, CD_3_CN) *δ*:149.8 149.2; HRMS (ESI^+^) m/z calculated for C_44_H_56_N_4_O_10_PS [M+H]^+^ 863.3410, found 863.3449.

**Solid‐phase synthesis of modified ORNs**: oligonucleotides were synthesized using an ABI model 394 DNA /RNA synthesizer on a 1 μmol scale using commercially available 2’‐*O*‐PivOM phosphoramidites (ChemGenes) and 2’‐*O*‐AcSM uridine phosphoramidite,^[9b]^ 2’‐*O*‐AcSE uridine phosphoramidite **4 a**, 2’‐*O*‐AcSP uridine phosphoramidite **4 b** and a long chain alkylamine controlled‐pore‐glass (LCAA‐CPG) as solid support. Oligonucleotides were assembled in TWIST^TM^ columns (Glen Research). Phosphoramidites were vacuum dried prior to their dissolution in extra dry acetonitrile (Biosolve) at 0.1 M concentration. Coupling for 180 s was performed with 5‐benzylmercaptotetrazole (BMT, 0.3 M) as the activator. The oxidizing solution was 0.1 M iodine in THF/pyridine/H20 (78 : 20 : 2, v/v/v). The capping step was performed with a mixture of 5 % phenoxyacetic anhydride in THF; Detritylation was performed with 3 % TCA in CH_2_Cl_2_. After RNA assembly completion, the column was removed from the synthesizer and dried under a steam of argon.

**Formation of 2’,2’‐disulfide bridged ON 1, H2, and H3**: After ORN elongation, the solid support inside the TWIST column was treated with 2 mL of 2,2’‐dithiopyridine (20 equiv.) in anhydrous BuNH_2_/THF (95 : 5 solution). The solution was applied to the synthesis column using two syringes with 4 A molecular sieve and was pushed back and forth though the synthesis column for 30 min. Then the solution was removed and the solid support was washed with anhydrous THF followed by 1 min flush with argon. Finally, the solid support was treated with a 30 % aqueous ammoniac solution for 3 h at 37 °C. The deprotection solution was evaporated in the presence of isopropylamine (13 % of total volume) under reduced pressure.

**Formation of 2’,2’‐disulfide bridged ON 2, ON 3 and H4**–**H7**: After ORN elongation, the solid support inside the TWIST column was treated with 2 mL of 10 % piperidine in anhydrous acetonitrile. The solution was applied to the synthesis column using two syringes with 4 A molecular sieve and was pushed back and forth though the synthesis column for 30 min. The solution was removed and the solid support was washed with anhydrous acetonitrile followed by 1 min flush with argon. Then, the solid support was treated with 2 mL of 2,2’‐dithiopyridine (20 equiv.) in anhydrous BuNH_2_/THF (95 : 5 solution). The solution was applied to the synthesis column using two syringes with 4 A molecular sieve and was pushed back and forth though the synthesis column for 30 min. Then the solution was removed and the solid support was washed with anhydrous THF followed by 1 min flush with argon. Finally, the solid support was treated with a 30 % aqueous ammoniac solution for 3 h at 37 °C. The deprotection solution was evaporated in the presence of isopropylamine (13 % of total volume) under reduced pressure.

**Purification**: The crudes ORNs were purified by semi‐preparative IEX‐HPLC with a linear gradient of buffer B in buffer A. The pure fractions of each ORN were pooled in a 100 mL round‐bottomed flask were concentrated to dryness under reduced pressure. The residues were dissolved in 10 mL of a 100 mM TEAAc solution and were loaded on a C18 cartridge (Waters, Sep‐Pak®) equilibrated with a 100 mM TEAAc solution. Elution was performed with a solution of 70 % CH_3_CN in water and the solution was concentrated to dryness under reduced pressure. Purified ORNs were analyzed by MALDI‐TOF MS and were quantified by UV measurements.

**Thermal denaturation experiments** *T_m_* experiments were performed using a CARY 300 UV Spectrophotometer (Varian Inc) equipped with a Peltier temperature controller and thermal analysis software. The sample were prepared by mixing ORN solutions to give 1.5 μM final concentration in 1 mL of buffer (10 mM Na Cacodylate, 100 mM NaCl, pH=7) in a 1 cm path length quartz cell. A heating‐cooling‐heating‐cooling cycle in the 5 °C–90 °C temperature range with a 0.5 °C.min^−1^ gradient was applied. *T_m_* values were determined from the maxima of the first derivative plots of the absorbance versus temperature. The *T_m_* values from two independent experiments were accurate within ±0.5 °C.

**CD Spectroscopy studies** CD spectra were recorded on a Jasco J‐815 spectropolarimeter. The solutions used for *T_m_* experiments were transferred into a 1 cm path length quartz cell. Measurements were performed at 1 °C with the wavelength range set to 340–200 nm with a scanning speed of 100 nm.min^−1^. Raw data were acquired over 2 scans.

**Enzymatic stability studies with snake venom phosphodiesterase (SVPDE)**: Aqueous solutions of ORN were prepared at concentration of 0.4 mM. Each ORN (10.5 μL, 4.2 nmol) was mixed with 4.2 μL of 450 mM citrate ammonium solution and 23.1 μL of water. SVPDE (4.2 μL/0.2 U/mL) was added and the mixture was incubated at 37 °C. At different incubation times, aliquots (6 μL) were withdrawn, immediately frozen and stored at −80 °C until analysis. Each sample was analyzed by IEX‐HPLC.

**Gel electrophoresis experiment**: 80 μM ORNs solutions were prepared in deionized water. For each ORN, aliquots were withdrawn, lyophilized and dissolved in 25 mM KCl, 2 mM MgCl_2_, and 25 mM Na‐cacodylate pH 7 (20 μL) to obtain 5 μM solutions. All samples were denatured by heating at 90 °C for 3–5 min followed by cooling to room temperature overnight; the samples were then kept at 4 °C for 3 h and diluted with formamide (20 μL). Each sample was analyzed by gel electrophoresis (15 % polyacrylamide, Tris borate MgCl_2_ (TBM) buffer 1X, 4 °C). The gels were stained with GelRed 3X (VWR) in a 0.1 M NaCl solution for 1 h and revealed using a UV transilluminator analysis. Band analysis was performed using Image J Software (Broken Symetry Ostware V: 1.4.3.67).

## Supporting Information

The data that support the findings of this study are available in the supplementary material of this article.

## Conflict of interests

There are no conflicts to declare.

1

## Supporting information

As a service to our authors and readers, this journal provides supporting information supplied by the authors. Such materials are peer reviewed and may be re‐organized for online delivery, but are not copy‐edited or typeset. Technical support issues arising from supporting information (other than missing files) should be addressed to the authors.

Supporting Information

## Data Availability

The data that support the findings of this study are available in the supplementary material of this article.
