# Supplementary material for: Synthesis and properties of RNA constrained by a 2’‐O‐disulfide bridge
Source: ChemistryOpen. 2024 Jan 10;13(8):e202300232. doi: 10.1002/open.202300232 (PMC11319213; doi:10.1002/open.202300232)
Supplement: Supplementary file 1 — Supporting Information [file OPEN-13-e202300232-s001.pdf]

ChemistryOpen

Supporting Information

# Synthesis and properties of RNA constrained by a 2′-*O*-disulfide bridge

Diallo Traoré, Elisa Biecher, Manon Mallet, Sonia Rouanet, Jean-Jacques Vasseur, Michael Smietana, and Christelle Dupouy*

## Contents

**Synthesis of 2-S-acetyl-*tert*-butyl-dimethylsilyl ethanol and 2-S-acetyl-*tert*-butyl-dimethylsilyl propanol** ........ **3**

**Data for the synthesized ORNs** ........ **4**

**Table S1**: Protocol for the formation of S-S bridge and MALDI-TOF data of ORNs ........ 4

**$^{1}$H, $^{13}$C, and $^{31}$P NMR spectra** ........ **5**

**Figure S1**: 400 MHz $^{1}$H-NMR spectrum ($CDCl_3$) of -bromoethoxy(*tert*-butyl)dimethylsilane 5

**Figure S2**: 400 MHz $^{1}$H-NMR spectrum ($CDCl_3$) of -3-bromopropoxy(*tert*-butyl)dimethylsilane ........ 5

**Figure S3**: 400 MHz $^{1}$H-NMR spectrum ($CDCl_3$) of S-acetyl-*tert*-butyl-dimethylsilyl ethanol ........ 6

**Figure S4**: 400 MHz $^{1}$H-NMR spectrum ($CDCl_3$) of S-acetyl-*tert*-butyl-dimethylsilyl propanol ........ 6

**Figure S5**: 400 MHz $^{1}$H-NMR and 100 MHz $^{13}$C-NMR spectra (DMSO-$d_6$) of **2a** ........ 7

**Figure S6**: 400 MHz $^{1}$H-NMR and 100 MHz $^{13}$C-NMR spectra (DMSO-$d_6$) of **2b** ........ 8

**Figure S7** : 400 MHz $^{1}$H-NMR and 100 MHz $^{13}$C-NMR spectra ($CDCl_3$) of **3a** ........ 9

**Figure S8**: 400 MHz $^{1}$H-NMR and 100 MHz $^{13}$C-NMR spectra ($CDCl_3$) of **3b** ........ 10

**Figure S9**: 400 MHz $^{1}$H-NMR and 121 MHz $^{31}$P-NMR spectra ($CD_3CN$) of **4a** ........ 11

**Figure S10:** 400 MHz $^{1}$H-NMR and 121 MHz $^{31}$P-NMR spectra ($CD_3CN$) of **4b** ........ 12

**HPLC chromatograms & MALDI-TOF spectra of purified oligoribonucleotides** ........ **13**

**Figure S11 :** IEX-HPLC and MALDI-TOF MS analysis of purified RNA **1** ........ 13

**Figure S12 :** IEX-HPLC and MALDI-TOF MS analysis of purified RNA **2** ........ 14

**Figure S13:** IEX-HPLC and MALDI-TOF MS analysis of purified RNA **3** ........ 15

**Figure S14:** IEX-HPLC and MALDI-TOF MS analysis of purified hairpin **H1** ........ 16

**Figure S15:** IEX-HPLC and MALDI-TOF MS analysis of purified hairpin **H2** ........ 17

**Figure S16:** IEX-HPLC and MALDI-TOF MS analysis of purified hairpin **H3**......................18

**Figure S17:** IEX-HPLC and MALDI-TOF MS analysis of purified hairpin **H4**......................19

**Figure S18:** IEX-HPLC and MALDI-TOF MS analysis of purified hairpin **H5**......................20

**Figure S19:** IEX-HPLC and MALDI-TOF MS analysis of purified hairpin **H6**......................21

**Figure S20:** IEX-HPLC and MALDI-TOF MS analysis of purified hairpin **H7**......................22

**Circular Dichroism spectra ........................................................................................ 23**

**Figure S21:** CD spectroscopy curves at 1°C of RNA/RNA duplexes **D1-D4** ......................23

**Figure S22:** CD spectroscopy curves at 1°C of RNA/DNA duplexes **D5-D8** ......................23

**Figure S23:** CD spectroscopy curves at 1°C of Hairpins **H1-H7** ........................................24

**Enzymatic stability of hairpin H3 ............................................................................... 25**

**Figure S24:** IEXP-HPLC analyses and MALDI-TOF MS spectra of hairpin **H3** incubated with SVPD at t = 120 min. .........................................................................................25

**Molecular docking simulation of dinucleotides UU ........................................................ 26**

**Figure S25 :** Molecular modeling of dinucleotides. ..............................................................26

**Table S2-:** Torsion angles α and ζ. and $C_{1'}$-$C_{1'i+}$distance of dinucleotides UU ....................26

## Synthesis of 2-S-acetyl-*tert*-butyl-dimethylsilyl ethanol and 2-S-acetyl-*tert*-butyl-dimethylsilyl propanol

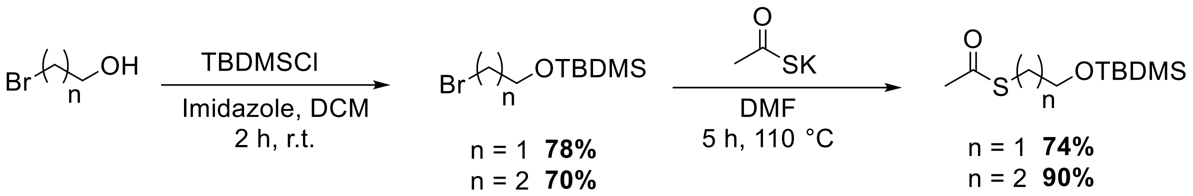


*The compounds are prepared as protocols previously reported.*[1]

*Step 1 General procedure: tert*-Butyldimethylsilyl chloride (2 equiv.) and imidazole (2 equiv.) were added to a solution of 2-bromoethanol (1 equiv.) or 3-bromopropanol (1 equiv.) in dichloromethane (1 mL / mmol) under argon and the colorless solution was stirred for 2 h at room temperature. The solution was diluted in dichloromethane, and washed successively with a saturated $NaHCO_3$ solution, water and brine, dried over $Na_2SO_4$ and concentrated *in vacuo*.

For 2-bromoethoxy(*tert*-butyl)dimethylsilane: purification through silica gel column chromatography (eluted in cyclohexane/acetone 10:1 v/v) gave the compound (78% yield) as a colorless liquid.$^1$H NMR (400 MHz, $CDCl_3$) $\delta$ : 3.88 (t, $J$ = 6.5 Hz, 2H), 3.37 (t, $J$ = 6.6 Hz, 2H), 0.90 (s, 9H), 0.08 (s, 6H).

For 3-bromopropoxy(*tert*-butyl)dimethylsilane: purification through silica gel column chromatography (eluted in ethylacetate/acetone 10:1 v/v) gave the compound (70% yield) as a colorless liquid.$^1$H NMR (400 MHz, $CDCl_3$) $\delta$ : 3.75 (t, $J$ = 6.0 Hz, 2H), 3.53 (t, $J$ = 6.5 Hz, 2H), 2.05 (quint. $J$ = 6.0 Hz, 2H), 0.91 (s, 9H), 0.08 (s, 6H).

*Step 2 General procedure:* 2-bromoethoxy(*tert*-butyl)dimethylsilane or -bromopropoxy(*tert*-butyl)dimethylsilane (1 equiv.) was diluted in anhydrous *N*,*N*dimethylformamide (1.3 mL / mmol) under argon at room temperature, and potassium thioacetate (1.3 equiv.) was added. The resulting slurry was heated to 110 °C for 5 h. The solvent was removed *in vacuo* and the residue was dissolved in ethyl acetate and washed with water. The aqueous phase was extracted with ethyl acetate (3 times). The organic layers were combined, washed with water (3 times), brine, dried over $Na_2SO_4$ and concentrated *in vacuo*.

For S-acetyl-*tert*-butyl-dimethylsilyl ethanol: purification through flash column chromatography (eluted in a gradient of ethyl acetate in cyclohexane 0-5%) gave the compound (74% yield) as a yellow liquid $^1$H NMR (400 MHz, $CDCl_3$) $\delta$: 3.66 (t, $J$ = 6.6 Hz, 2H), 2.97 (t, $J$ = 6.6 Hz, 2H), 2.27 (s, 3H), 0.84 (s, 9H), 0.02 (s, 6H).

For 2-S-acetyl-*tert*-butyl-dimethylsilyl propanol: purification through flash column chromatography (eluted in hexane/ethylacetate 10:1 v/v) gave the compound (90% yield) as a colorless liquid. $^1$H NMR (400 MHz, $CDCl_3$) $\delta$: 3.65 (t, $J$ = 6.0 Hz, 2H), 2.98 (t, $J$ = 6.5 Hz, 2H), 2.32 (s, 3H), 1.78 (quint. $J$ = 6.1 Hz, 2H), 0.89 (s, 9H), 0.04 (s, 6H).

[1] Sergev, D. Poly(ether-thioether)-, poly(ether-sulfoxide)-, and poly(ether-sulfone) nucleic acids, their synthesis and use in medicine and biochemistry. US6348583, 2002

## Data for the synthesized ORNs

| ORN | RNA Sequences[a] | Protocol for the formation of the disulfide bridge[b] | MALDI-TOF MS[c] | |
|---|---|---|---|---|
| | | | Calcd. | Found |
| **ON1** | 5'GCC UUC **$U_1U_1$**A UGA UU 3' | A | 4434.73 | 4434.56 |
| **ON2** | 5'GCC UUC **$U_2U_2$**A UGA UU 3' | B | 4462.79 | 4462.46 |
| **ON3** | 5'GCC UUC **$U_3U_3$**A UGA UU 3' | B | 4490.84 | 4490.12 |
| **H2** | 5'AUCCAG**$U_1U_1$**UUUGGAU 3' | A | 4805.99 | 4805.61 |
| **H3** | 5'AUCCAGU**$U_1U_1$**UUGGAU 3' | A | 4805.99 | 4805.13 |
| **H4** | 5'AUCCAG**$U_2U_2$**UUUGGAU 3' | B | 4832.04 | 4832.70 |
| **H5** | 5'AUCCAGU**$U_2U_2$**UUGGAU 3' | B | 4832.04 | 4833.61 |
| **H6** | 5'AUCCAG**$U_3U_3$**UUUGGAU 3' | B | 4861.13 | 4861.02 |
| **H7** | 5'AUCCAGU**$U_3U_3$**UUGGAU 3' | B | 4861.13 | 4861.58 |

[a] **$U_1U_1$** = 2',2' DME S-S bridge; **$U_2U_2$** = 2',2' DEE S-S bridge, **$U_3U_3$** = 2',2' DPE S-S bridge. [b]**Protocol A** : dithiopyridine (20 equiv.), $BuNH_2$/THF 5/95, 30 min, r.t. **Protocol B** : 1) 10% piperidine in ACN, 30 min, rt. 2) dithiopyridine (20 equiv.), $BuNH_2$/THF 5/95, 30 min, rt. [c]negative mode.

**Table S1**: Protocol for the formation of S-S bridge and MALDI-TOF data of ORNs

## $^{1}H$, $^{13}C$, and $^{31}P$ NMR spectra

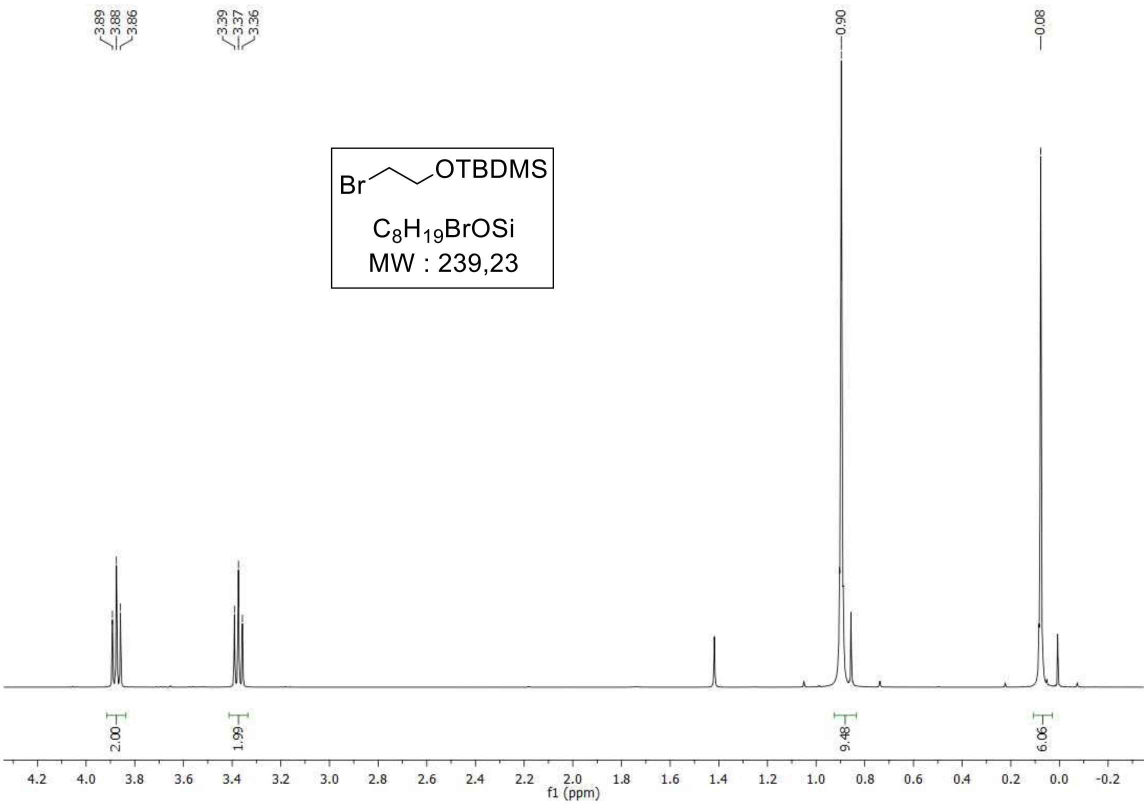


**Figure S1**: 400 MHz $^{1}$H-NMR spectrum ($CDCl_3$) of -bromoethoxy(*tert*-butyl)dimethylsilane

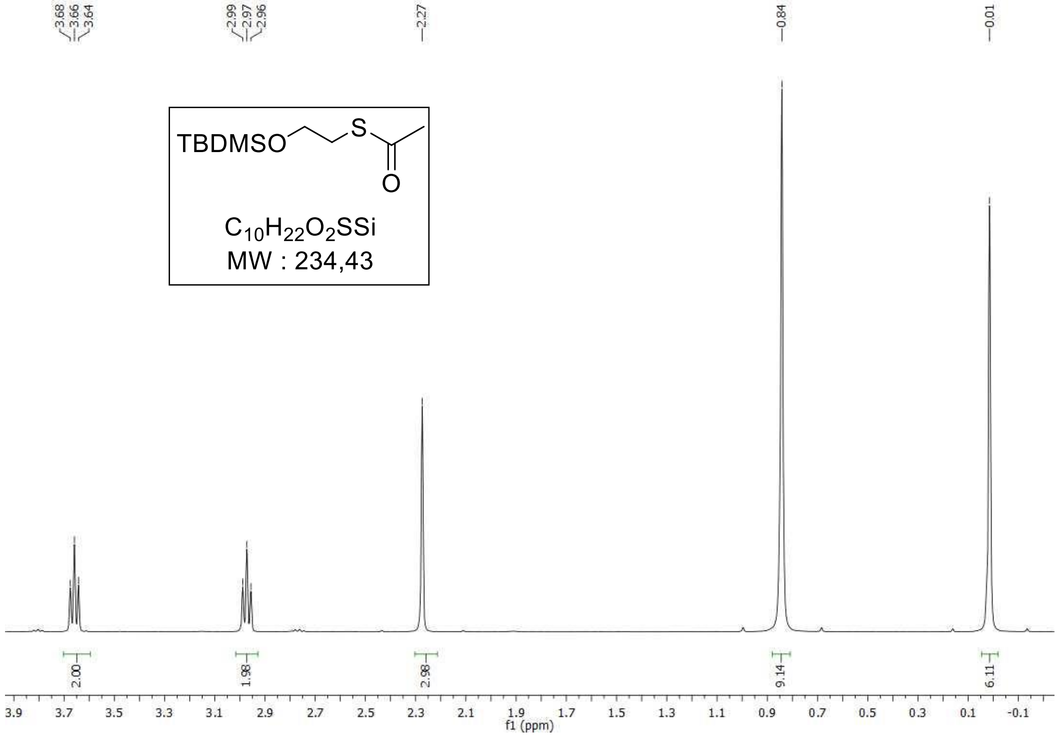


**Figure S2**: 400 MHz $^{1}$H-NMR spectrum ($CDCl_3$) of -3-bromopropoxy(*tert*-butyl)dimethylsilane

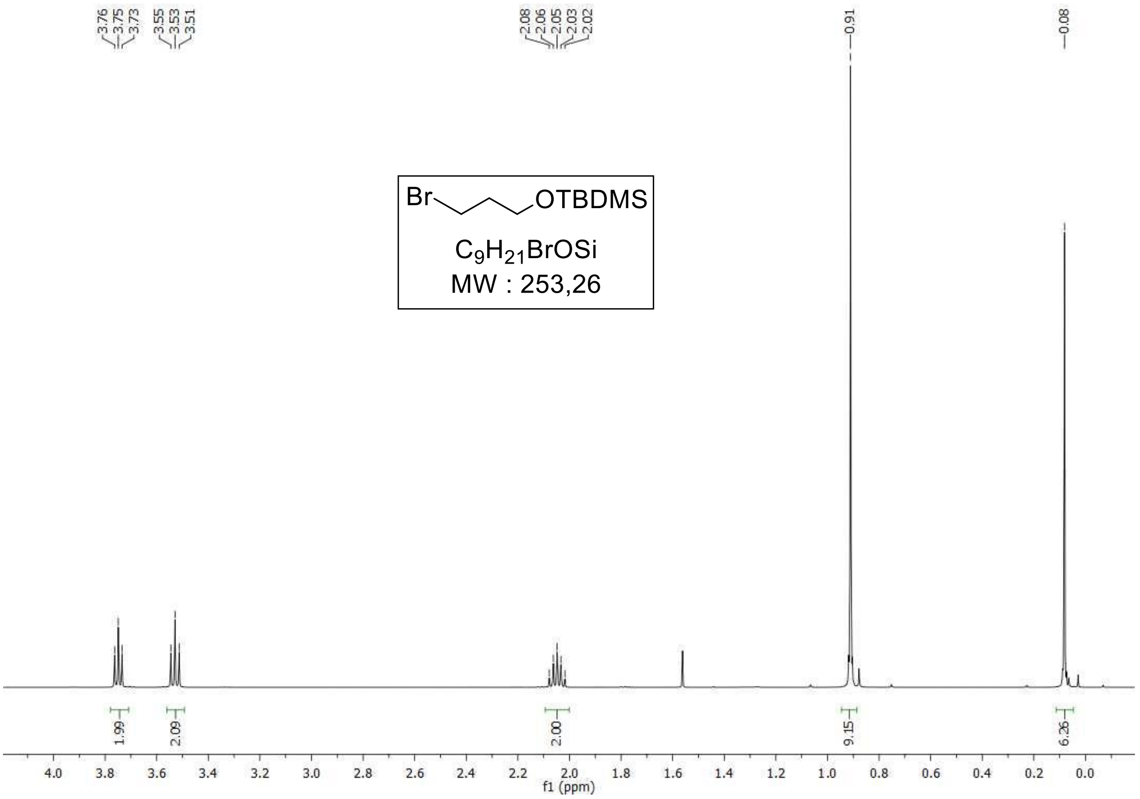


**Figure S3**: 400 MHz $^1$H-NMR spectrum ($CDCl_3$) of S-acetyl-*tert*-butyl-dimethylsilyl ethanol

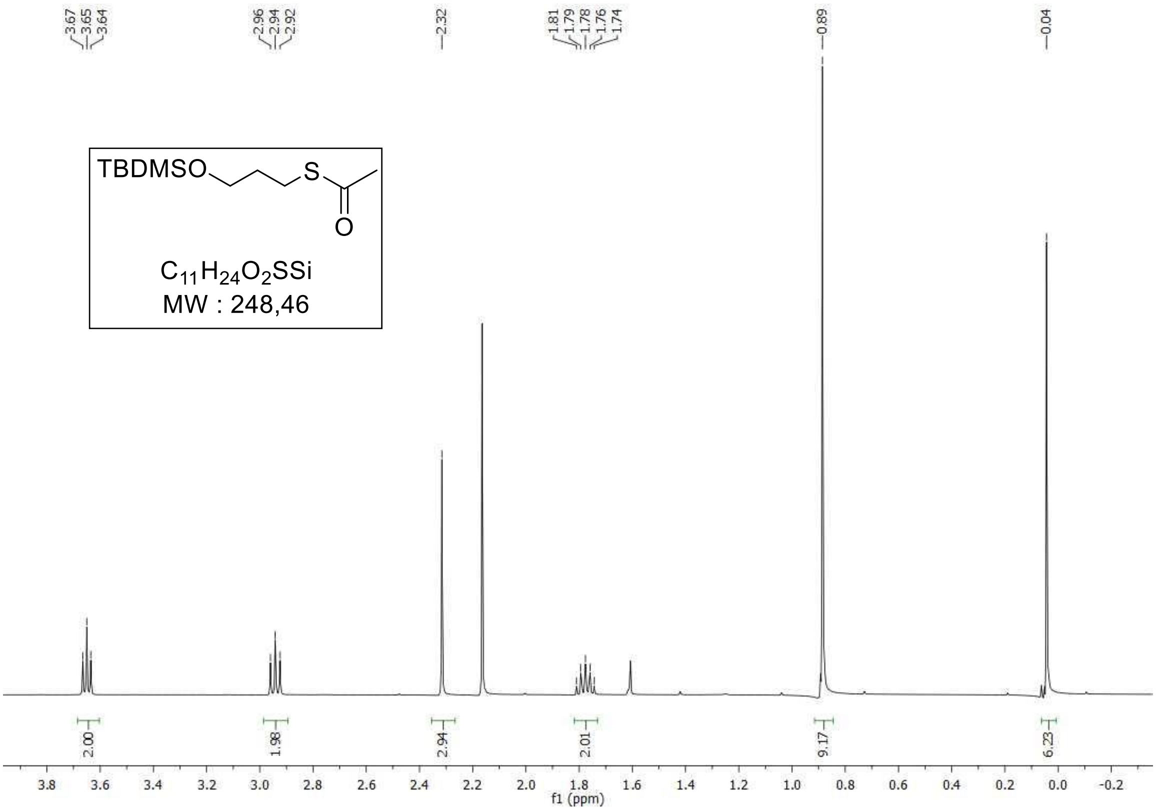


**Figure S4**: 400 MHz $^1$H-NMR spectrum ($CDCl_3$) of S-acetyl-*tert*-butyl-dimethylsilyl propanol

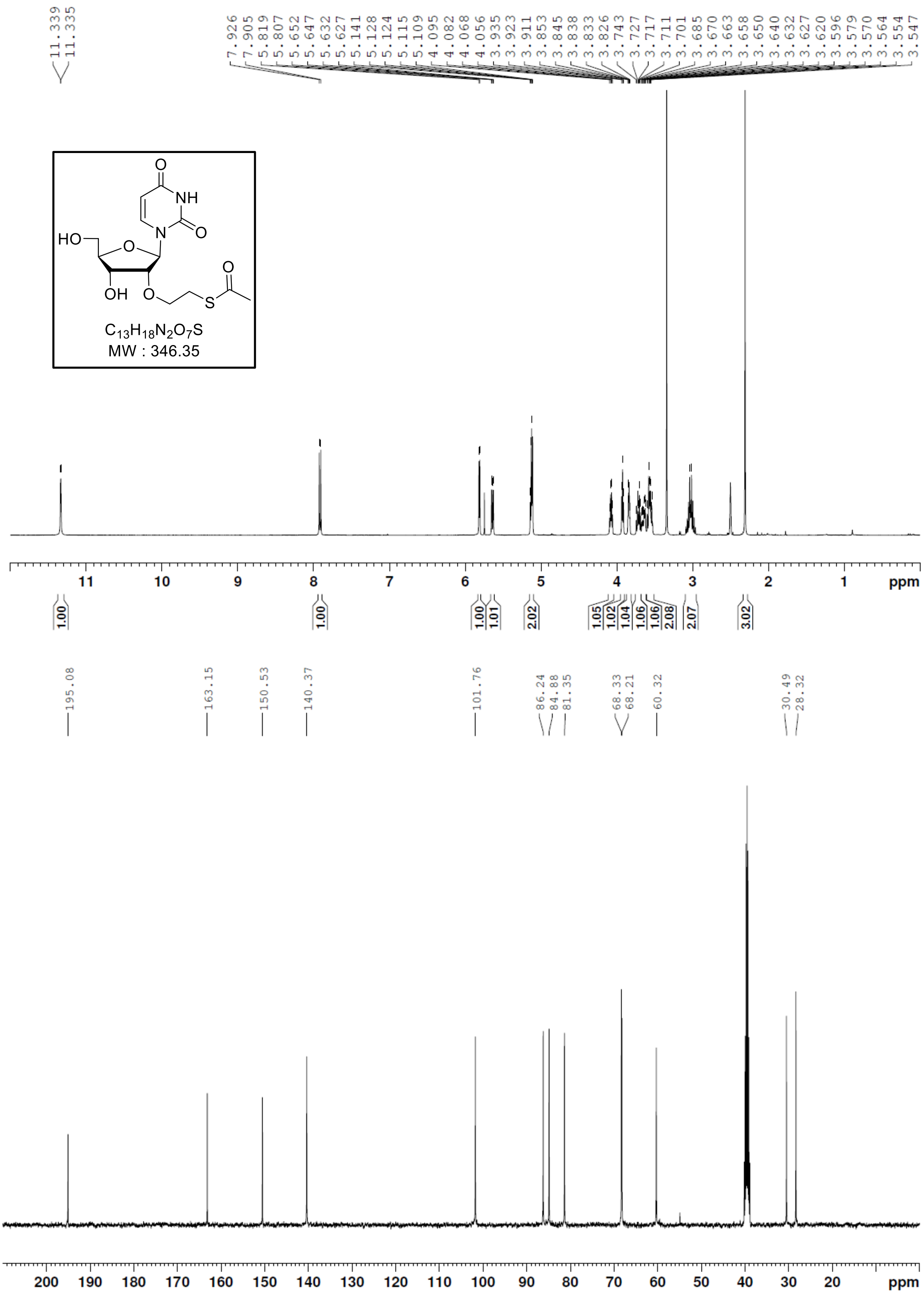


**Figure S5**: 400 MHz $^{1}$H-NMR and 100 MHz $^{13}$C-NMR spectra (DMSO-$d_6$) of **2a**

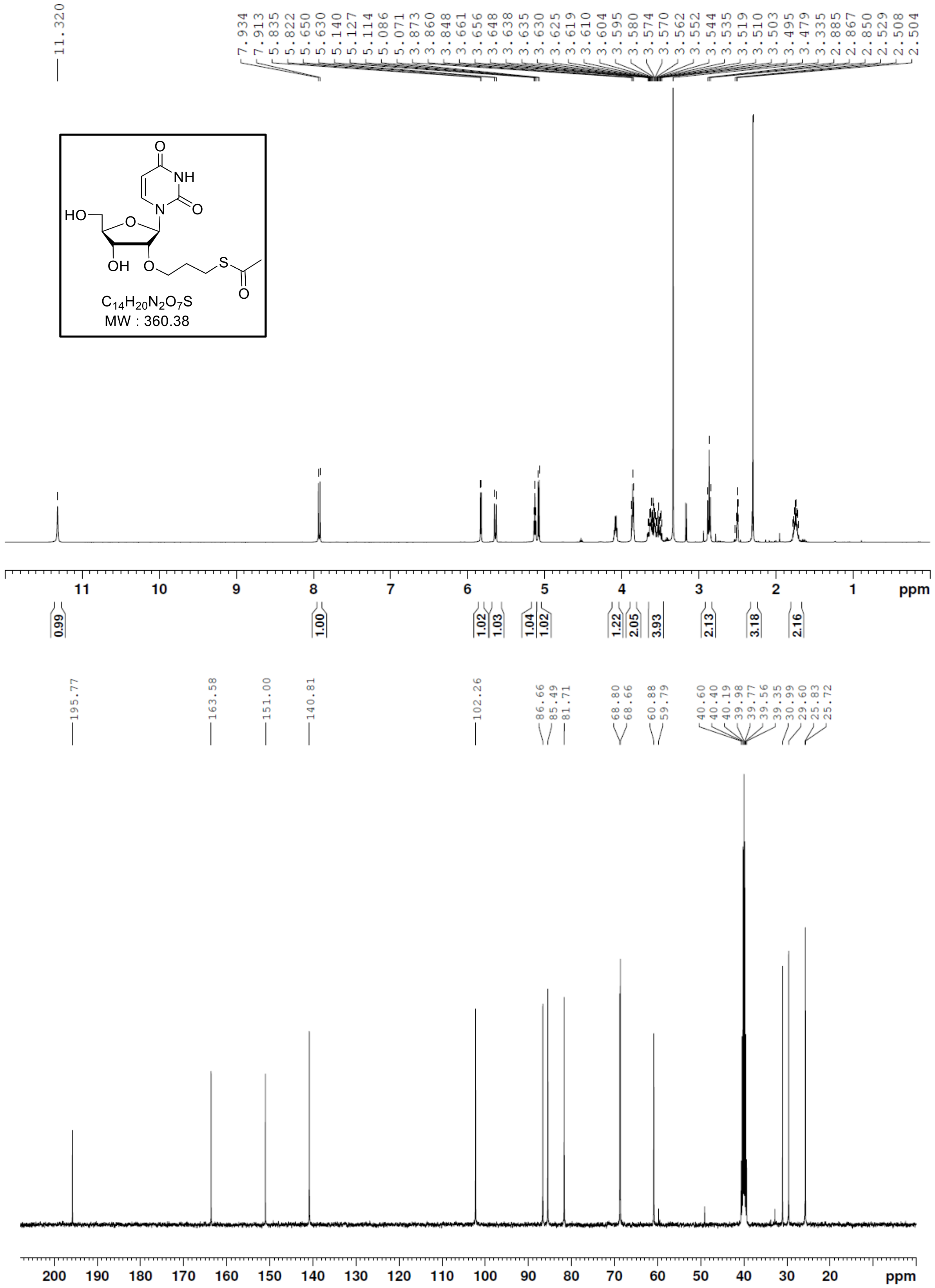


**Figure S6**: 400 MHz $^{1}H$-NMR and 100 MHz $^{13}C$-NMR spectra (DMSO-$d_6$) of **2b**

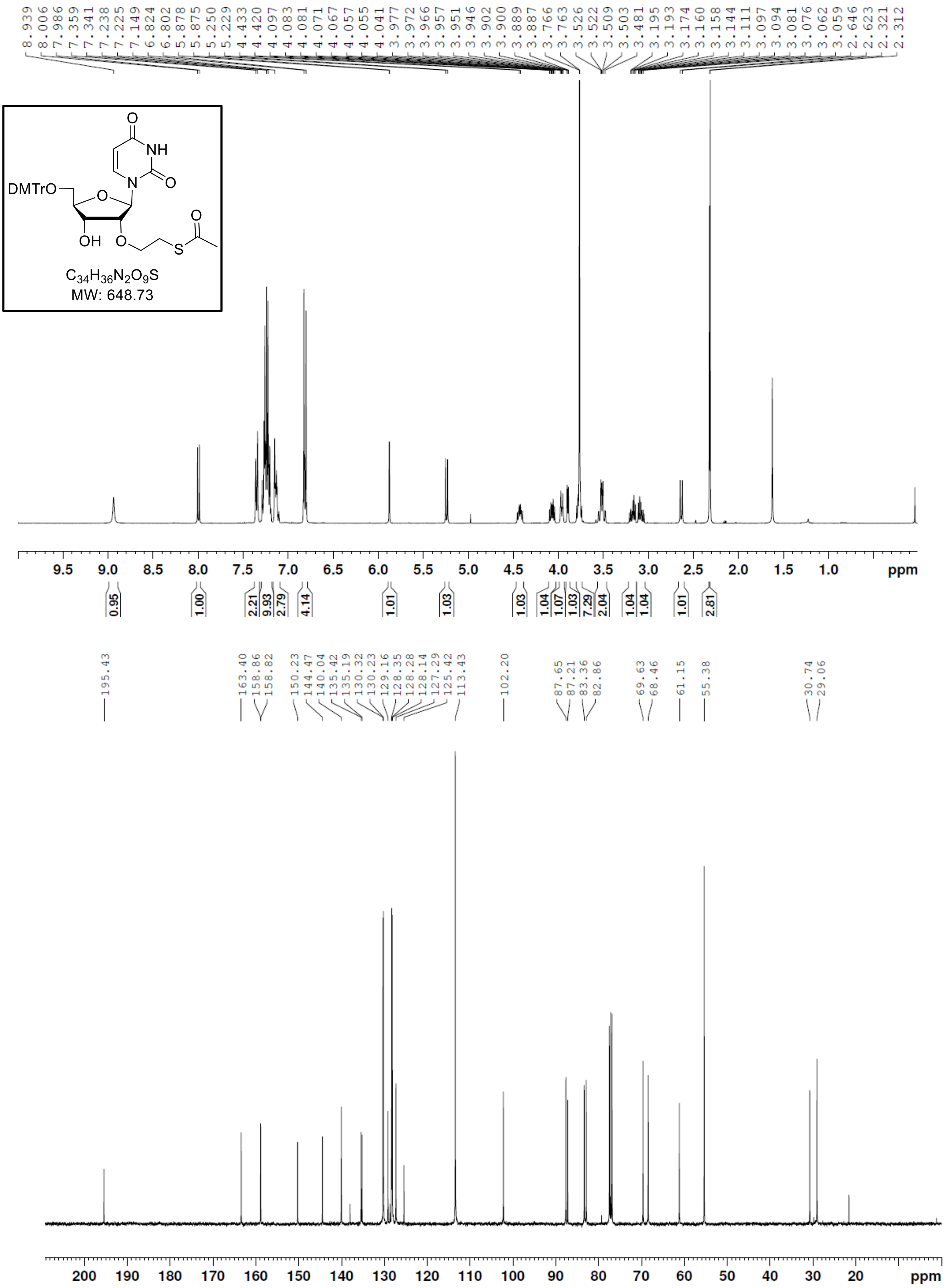


**Figure S7** : 400 MHz $^{1}$H-NMR and 100 MHz $^{13}$C-NMR spectra ($CDCl_3$) of **3a**

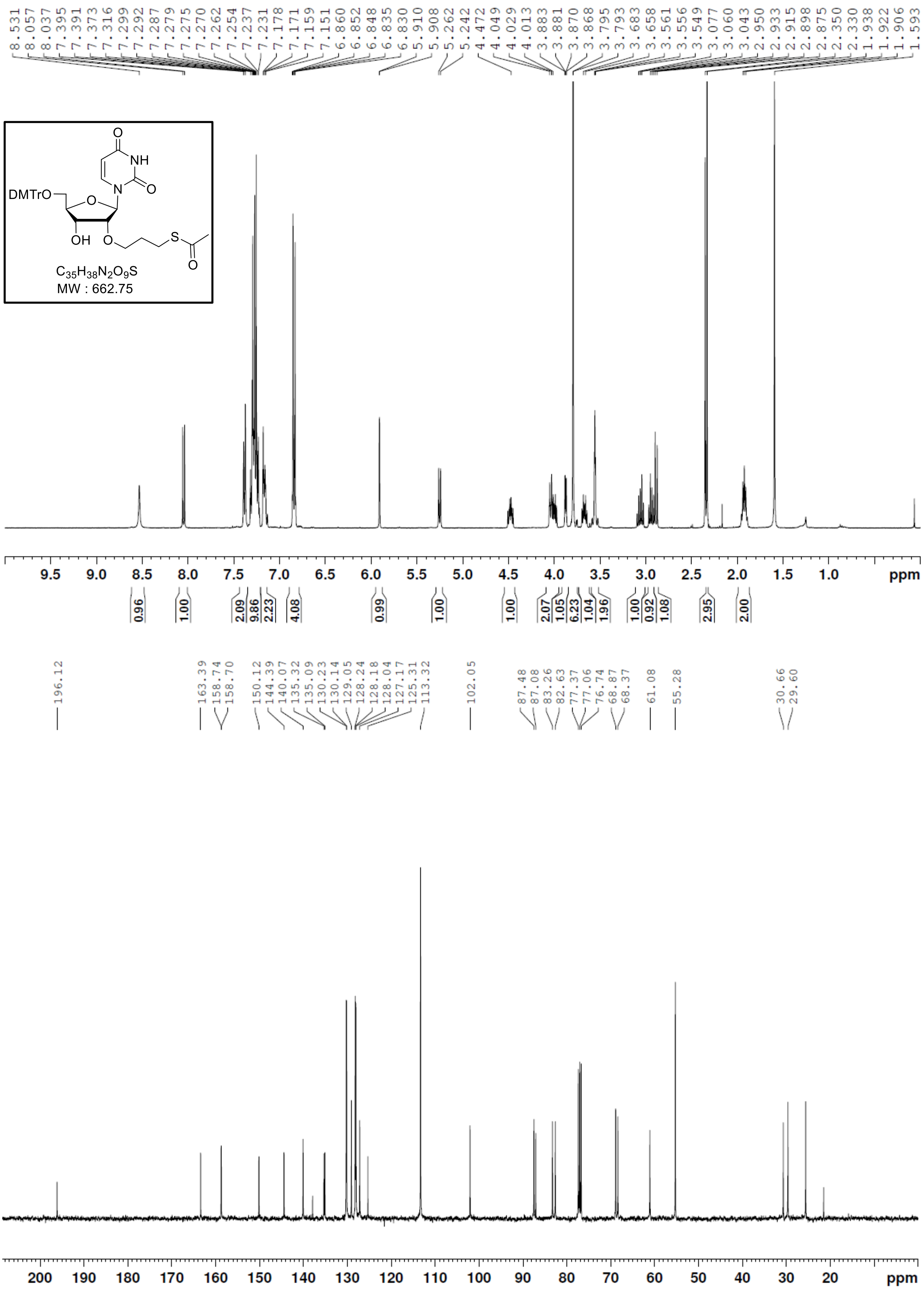


**Figure S8**: 400 MHz $^1$H-NMR and 100 MHz $^{13}$C-NMR spectra ($CDCl_3$) of **3b**

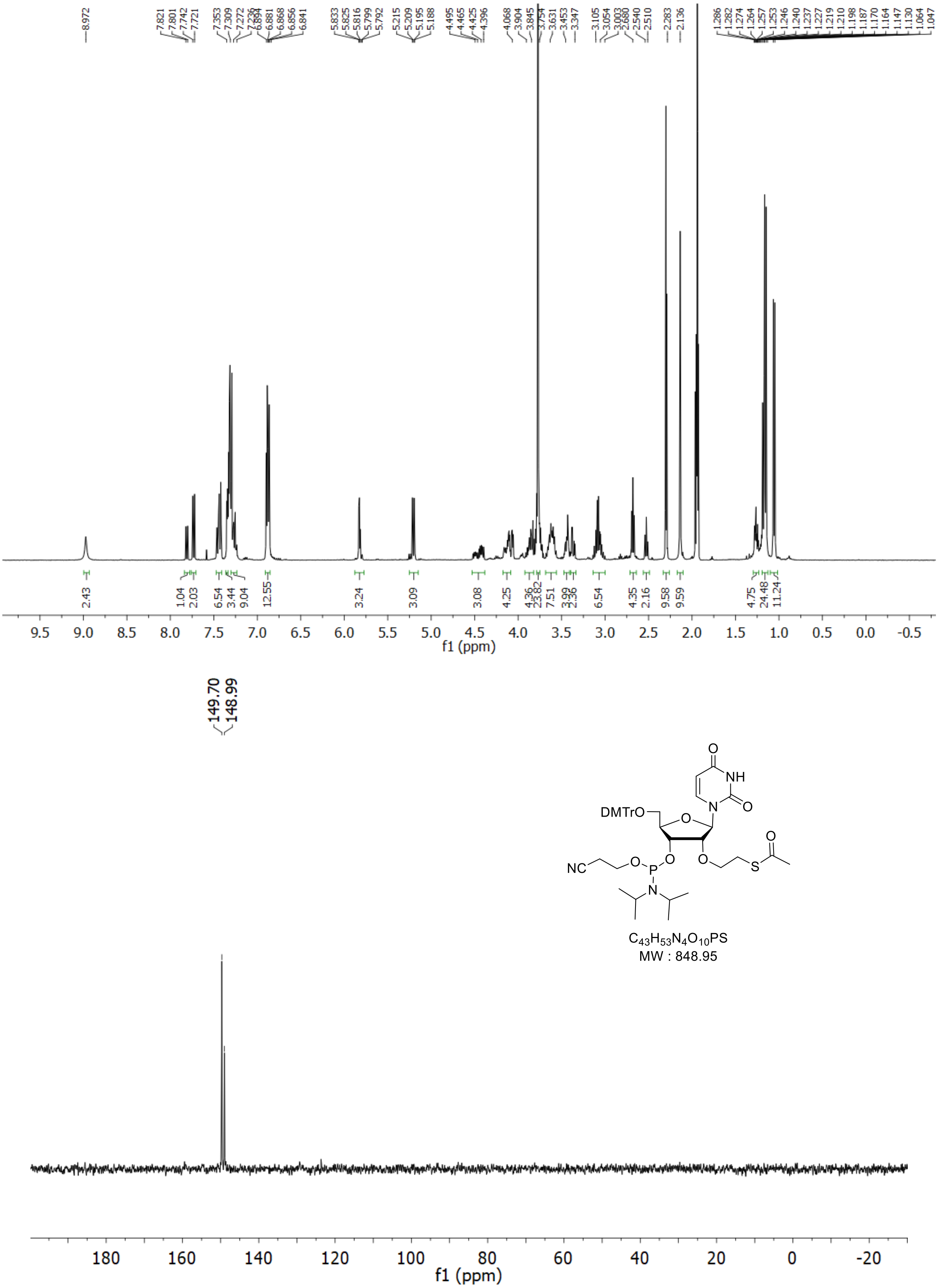


**Figure S9**: 400 MHz $^{1}H$-NMR and 121 MHz $^{31}P$-NMR spectra ($CD_3CN$) of **4a**

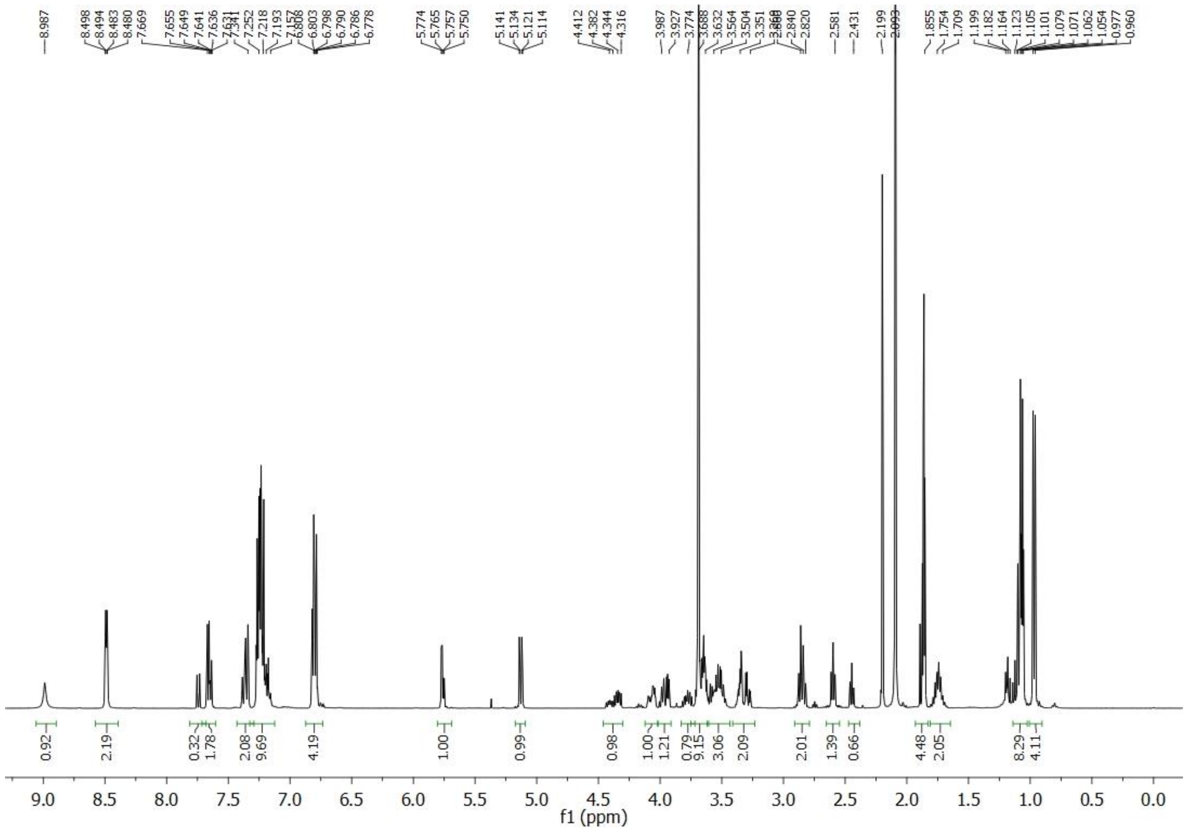


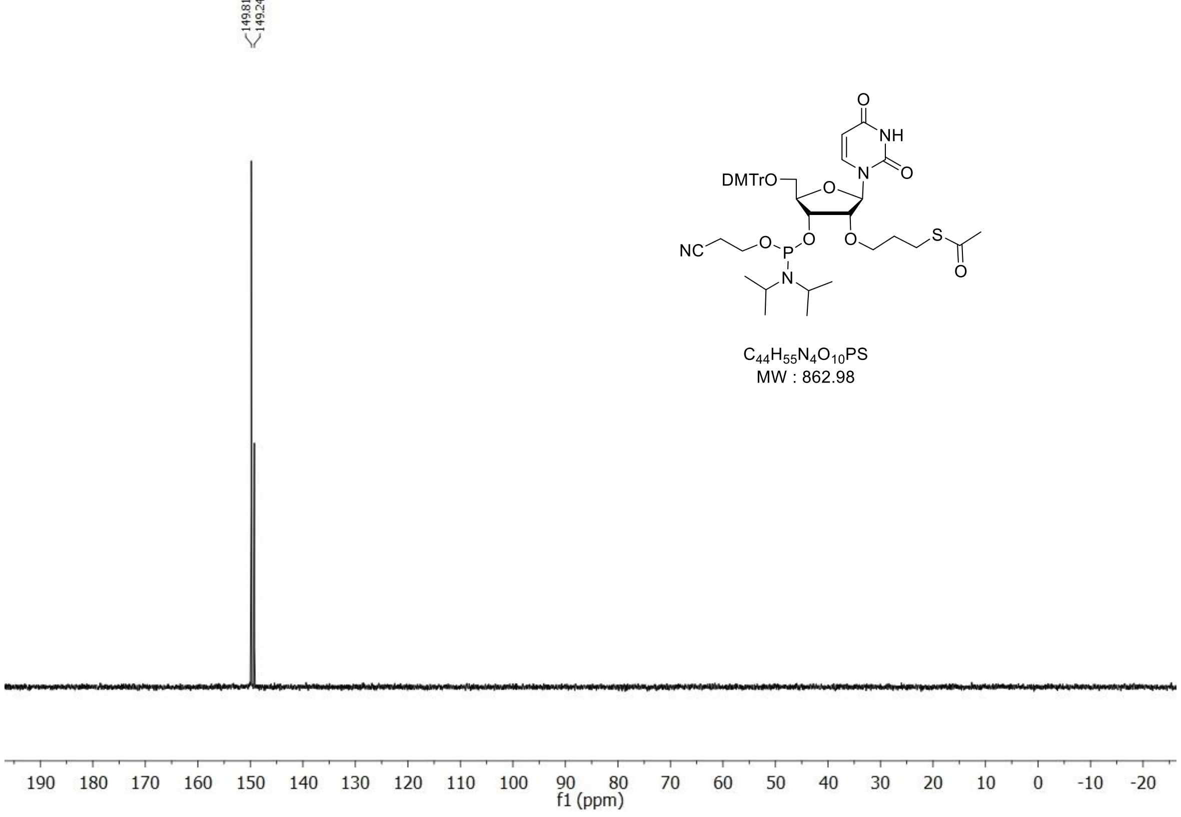


$C_{44}H_{55}N_4O_{10}PS$
MW : 862.98

**Figure S10:** 400 MHz $^{1}$H-NMR and 121 MHz $^{31}$P-NMR spectra ($CD_3CN$) of **4b**

## HPLC chromatograms & MALDI-TOF spectra of purified oligoribonucleotides

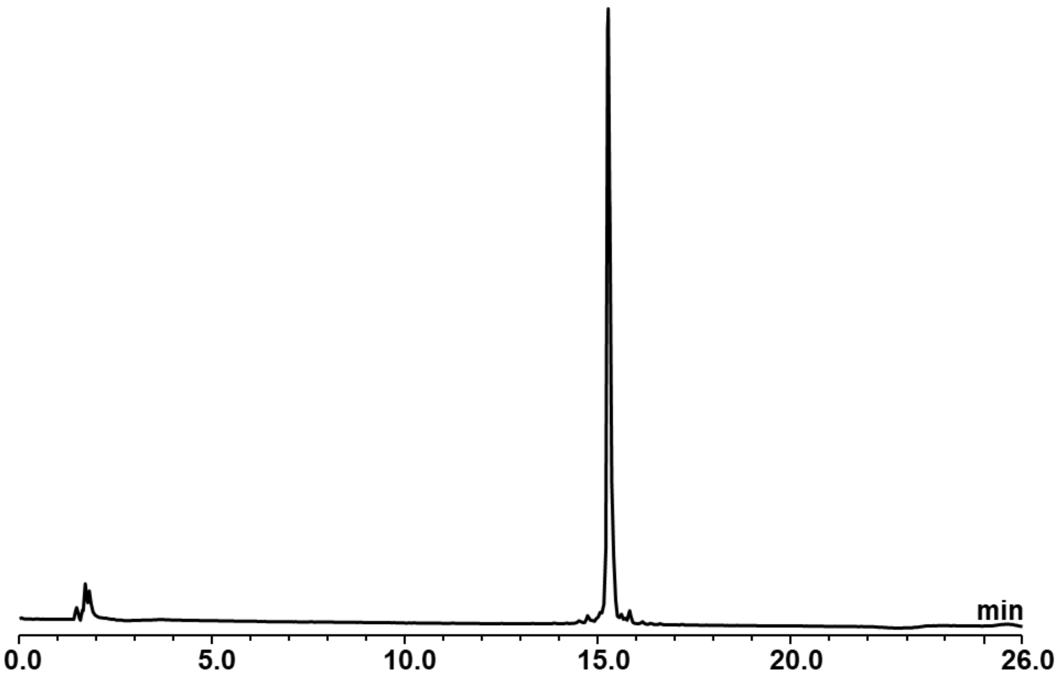


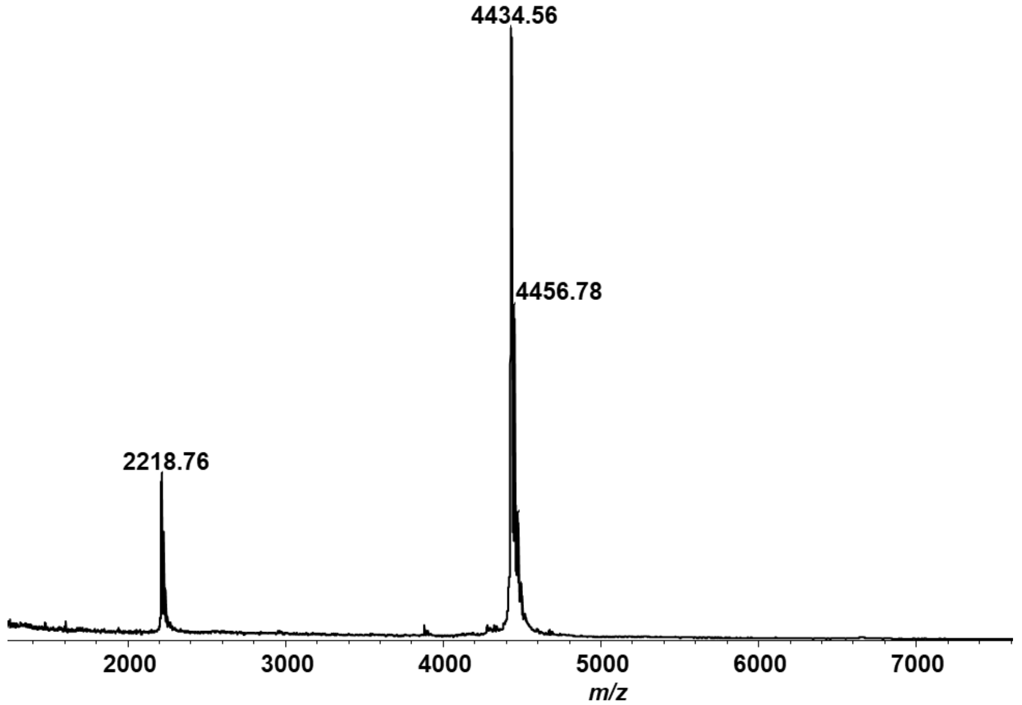


**Figure S11 :** IEX-HPLC and MALDI-TOF MS analysis of purified RNA **1**

*IEX-HPLC analysis conditions: DNAPac® PA100, 4X250 mm, elution with a 20 min linear gradient of 5 to 30% of B in eluent A. Column temperature 30°C. Flow rate 1.0 mL.min*$^{-1}$*. λ 260 nm.*
*Eluant A : 25 mM Tris HCl, 5% ACN, pH 8*
*Eluant B : 25 mM Tris HCl, 400 mM* $NaClO_4$*, 5% ACN, pH 8*

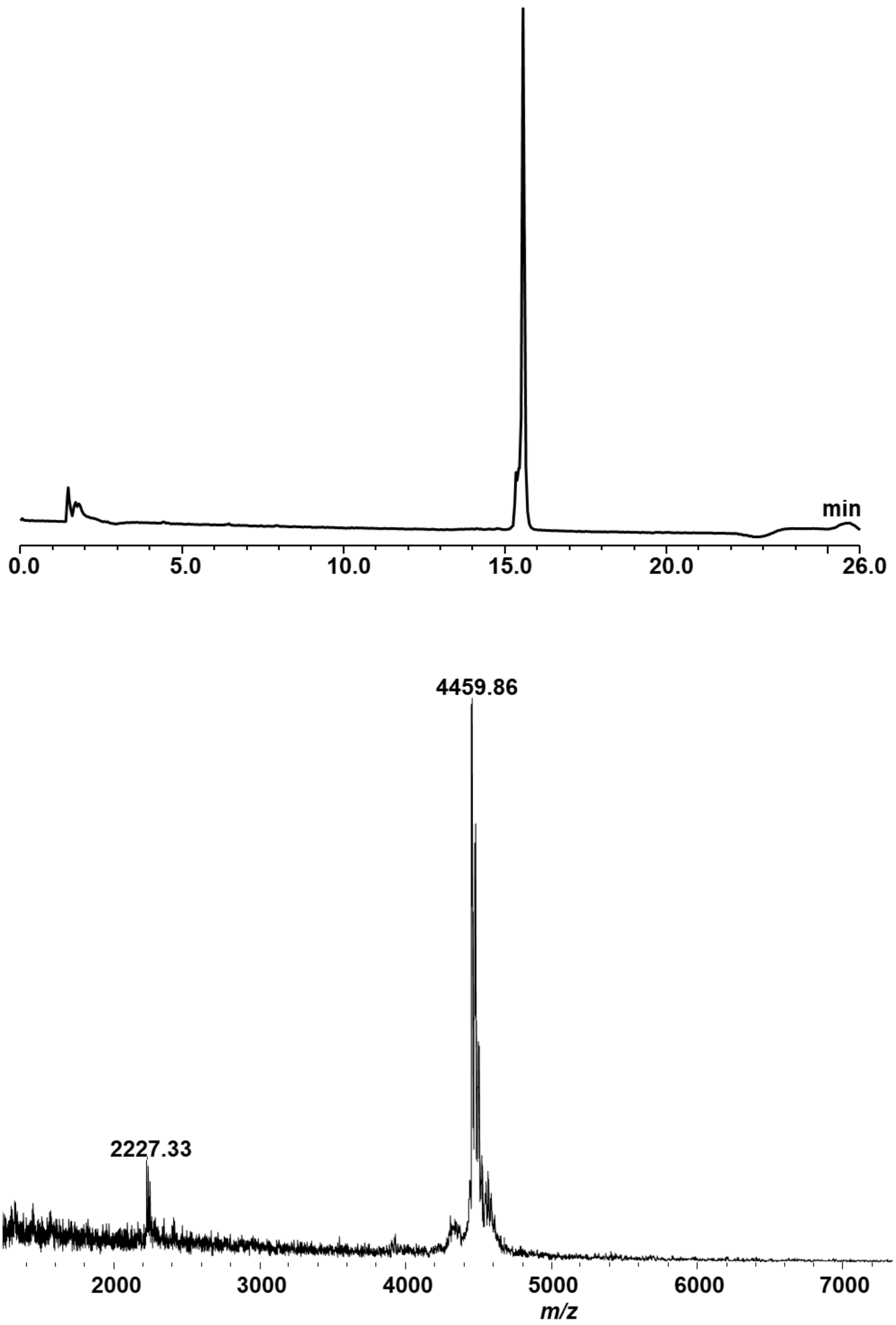


**Figure S12 :** IEX-HPLC and MALDI-TOF MS analysis of purified RNA **2**

*IEX-HPLC analysis conditions: DNAPac® PA100, 4X250 mm, elution with a 20 min linear gradient of 5 to 30% of B in eluent A. Column temperature 30°C. Flow rate 1.0 mL.min$^{-1}$. λ 260 nm.*
*Eluant A : 25 mM Tris HCl, 5% ACN, pH 8*
*Eluant B : 25 mM Tris HCl, 400 mM $NaClO_4$, 5% ACN, pH 8*

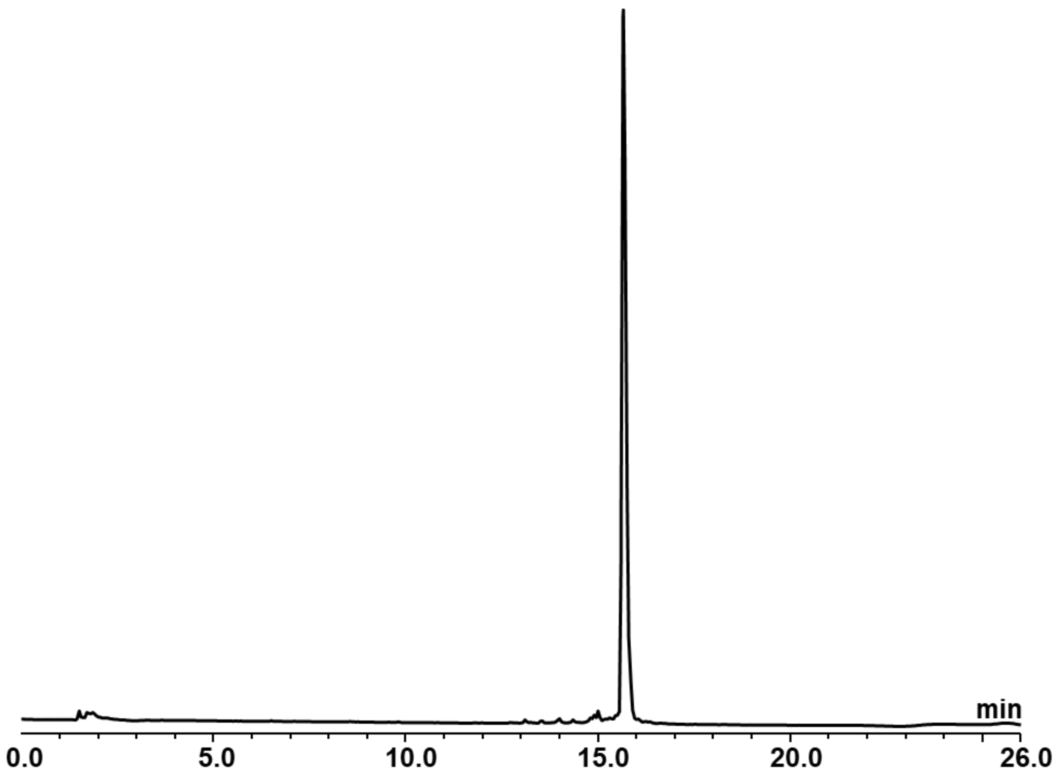


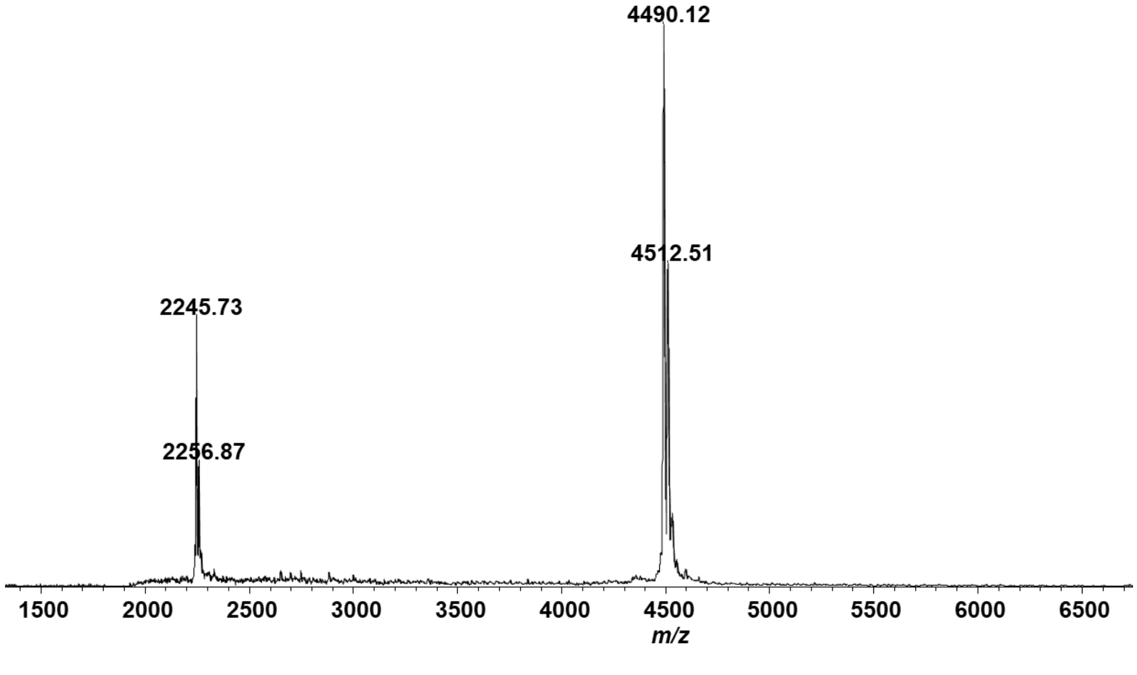


**Figure S13:** IEX-HPLC and MALDI-TOF MS analysis of purified RNA **3**

*IEX-HPLC analysis conditions: DNAPac® PA100, 4X250 mm, elution with a 20 min linear gradient of 5 to 30% of B in eluent A. Column temperature 30°C. Flow rate 1.0 mL.min$^{-1}$. λ 260 nm.*
*Eluant A : 25 mM Tris HCl, 5% ACN, pH 8*
*Eluant B : 25 mM Tris HCl, 400 mM $NaClO_4$, 5% ACN, pH 8*

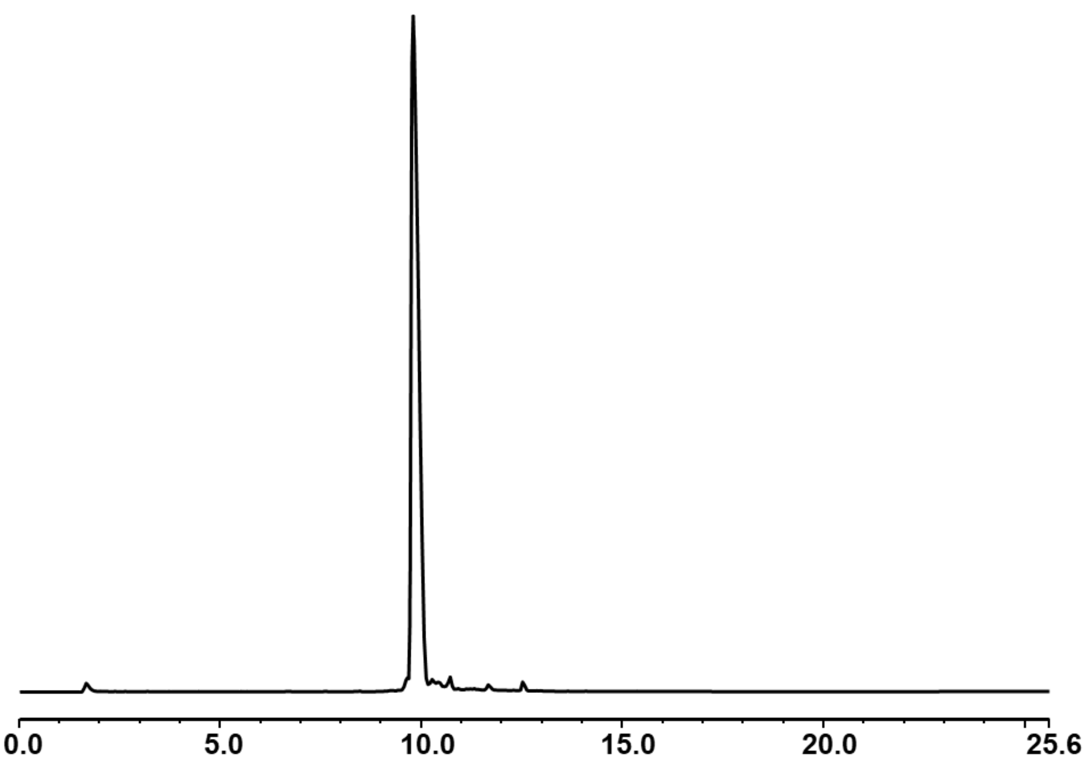


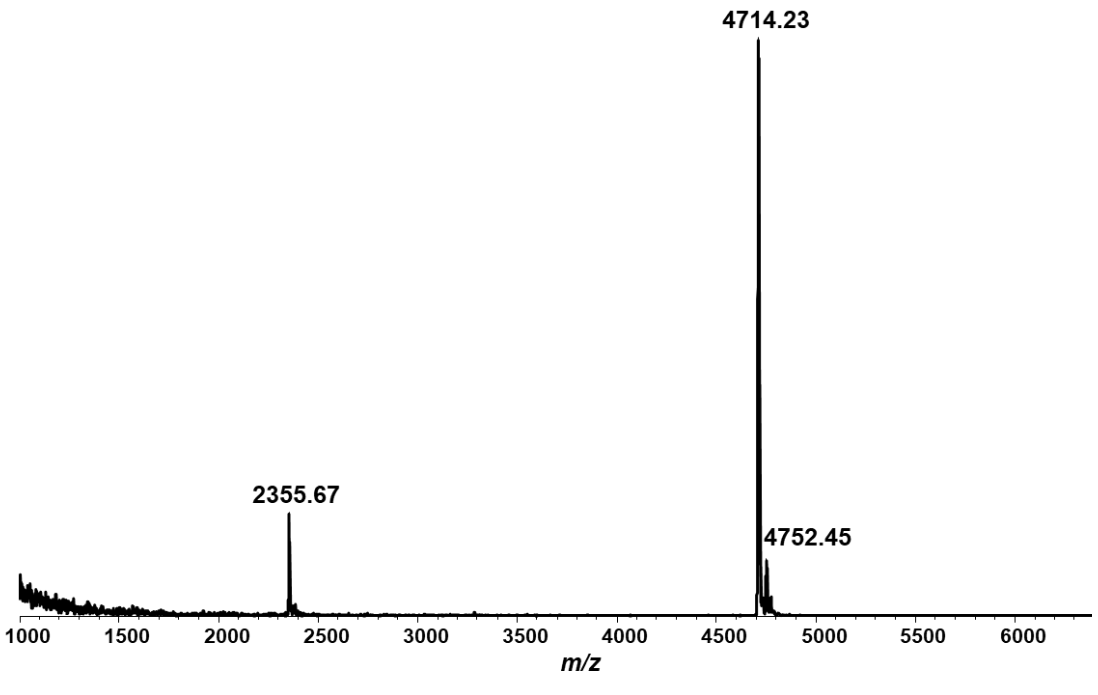


**Figure S14:** IEX-HPLC and MALDI-TOF MS analysis of purified hairpin **H1**

*IEX-HPLC analysis conditions: DNAPac® PA100, 4X250 mm, elution with a 20 min linear gradient of 10 to 50% of B in eluent A. Column temperature 30°C. Flow rate 1.0 mL.min$^{-1}$. λ 260 nm.*
*Eluant A : 25 mM Tris HCl, 5% ACN, pH 8*
*Eluant B : 25 mM Tris HCl, 400 mM $NaClO_4$, 5% ACN, pH 8*

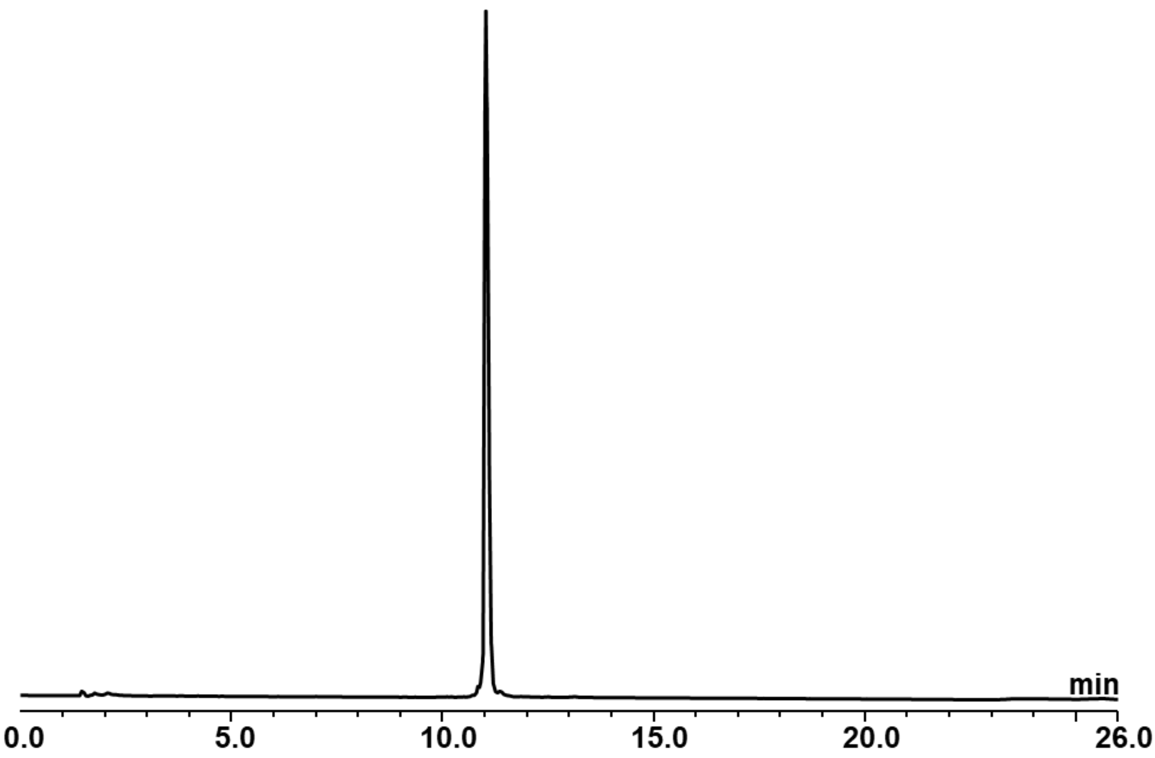


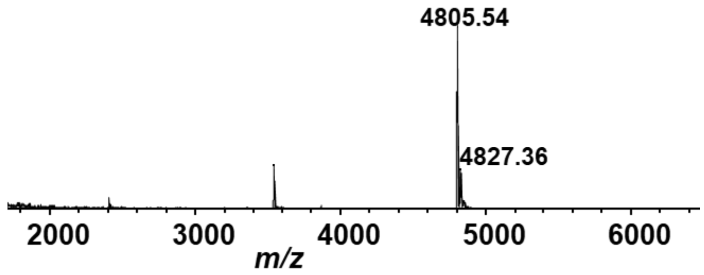


**Figure S15:** IEX-HPLC and MALDI-TOF MS analysis of purified hairpin **H2**

*IEX-HPLC analysis conditions: DNAPac® PA100, 4X250 mm, elution with a 20 min linear gradient of10 to 50% of B in eluent A. Column temperature 30°C. Flow rate 1.0 mL.min$^{-1}$. λ 260 nm.*
*Eluant A : 25 mM Tris HCl, 5% ACN, pH 8*
*Eluant B : 25 mM Tris HCl, 400 mM $NaClO_4$, 5% ACN, pH 8*

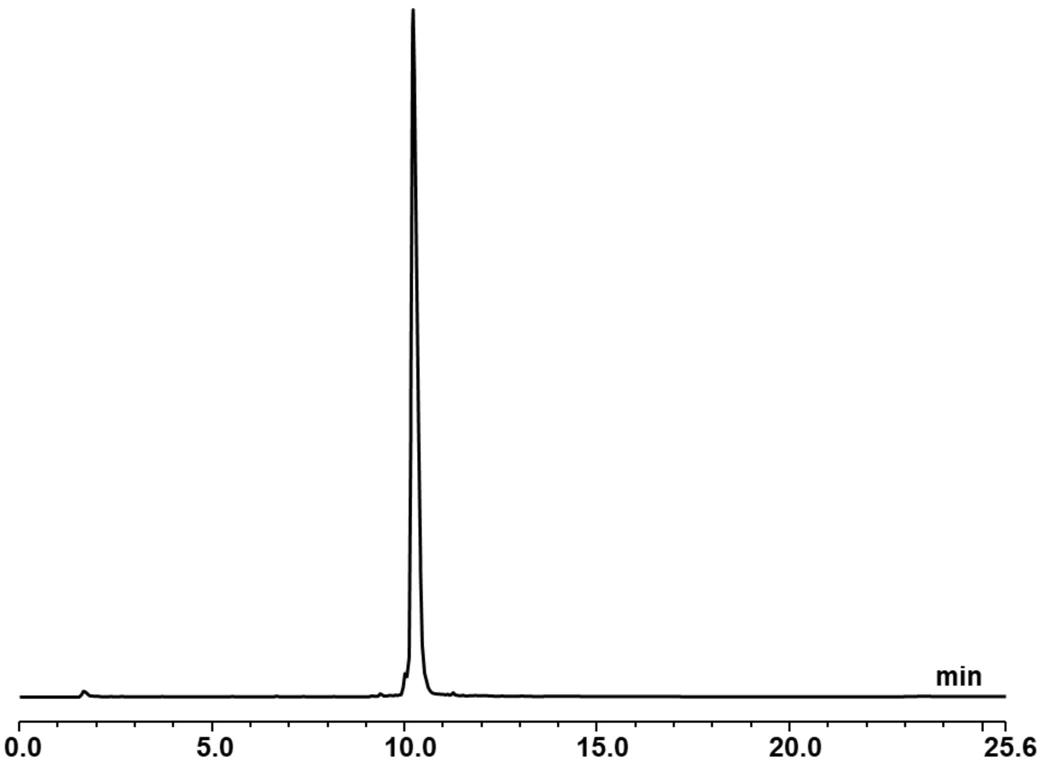


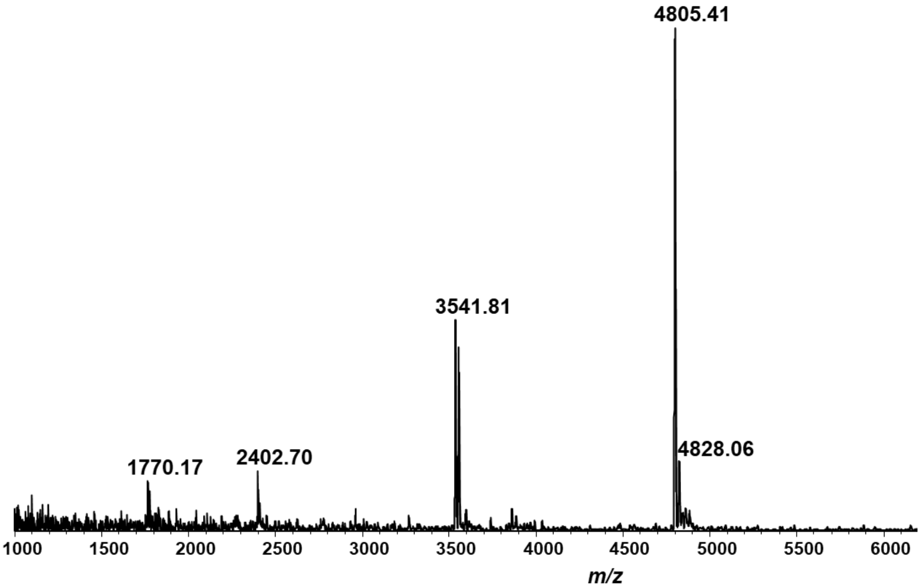


**Figure S16:** IEX-HPLC and MALDI-TOF MS analysis of purified hairpin **H3**

*IEX-HPLC analysis conditions: DNAPac® PA100, 4X250 mm, elution with a 20 min linear gradient of10 to 50% of B in eluent A. Column temperature 30°C. Flow rate 1.0 mL.min$^{-1}$. λ 260 nm.*
*Eluant A : 25 mM Tris HCl, 5% ACN, pH 8*
*Eluant B : 25 mM Tris HCl, 400 mM $NaClO_4$, 5% ACN, pH 8*

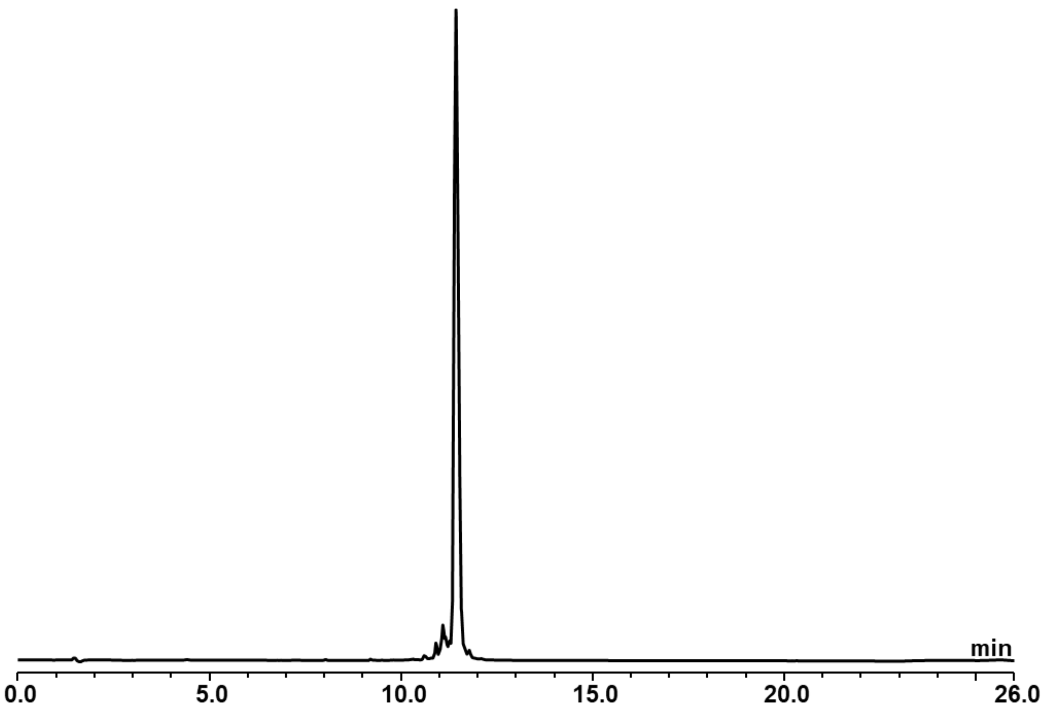


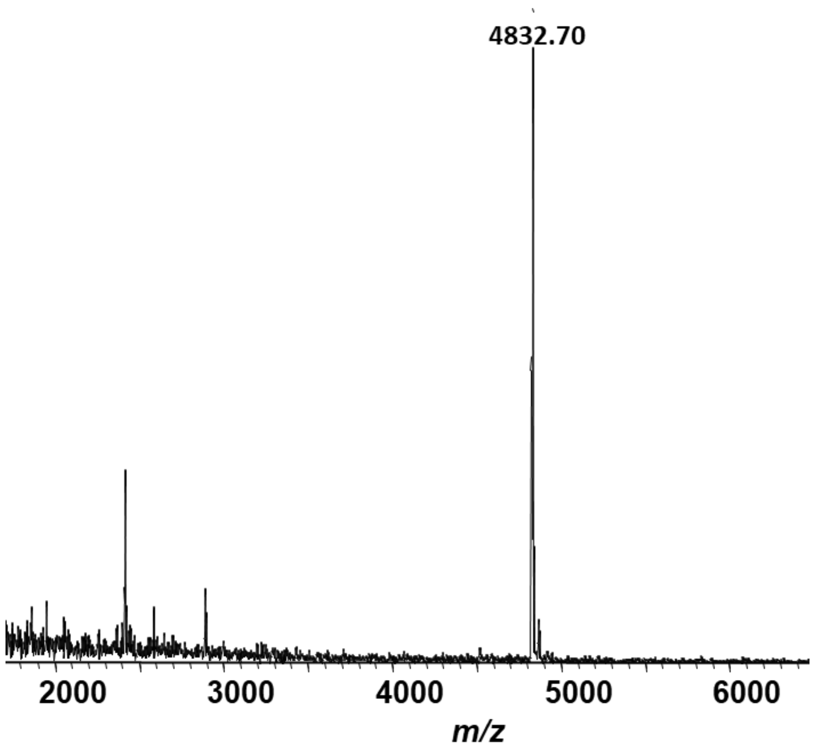


**Figure S17:** IEX-HPLC and MALDI-TOF MS analysis of purified hairpin **H4**

*IEX-HPLC analysis conditions: DNAPac® PA100, 4X250 mm, elution with a 20 min linear gradient of 5 to 40% of B in eluent A. Column temperature 30°C. Flow rate 1.0 mL.min*$^{-1}$*. λ 260 nm.*
*Eluant A : 25 mM Tris HCl, 5% ACN, pH 8*
*Eluant B : 25 mM Tris HCl, 400 mM* $NaClO_4$*, 5% ACN, pH 8*

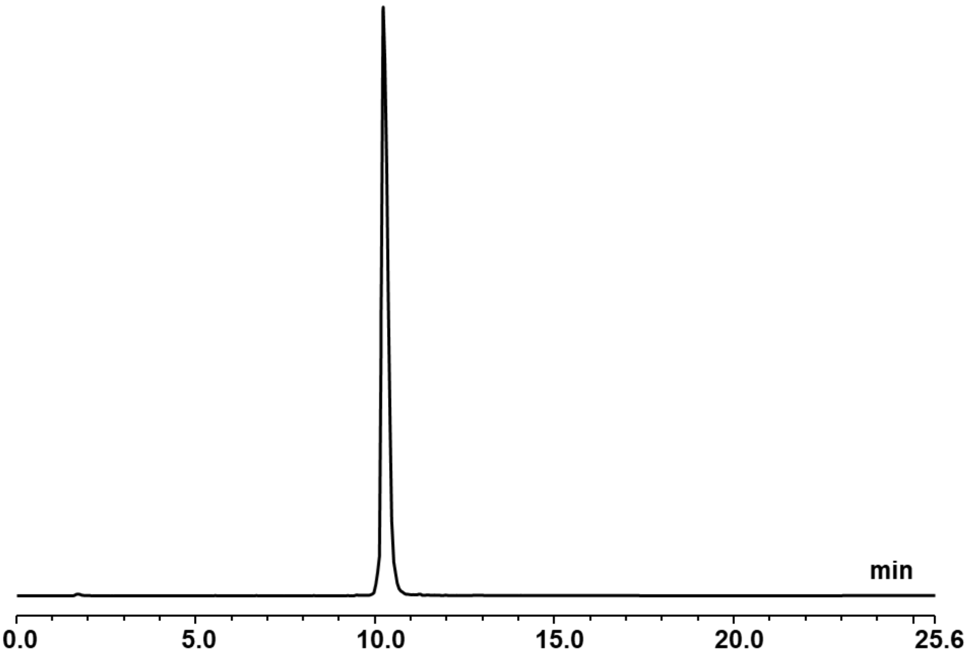


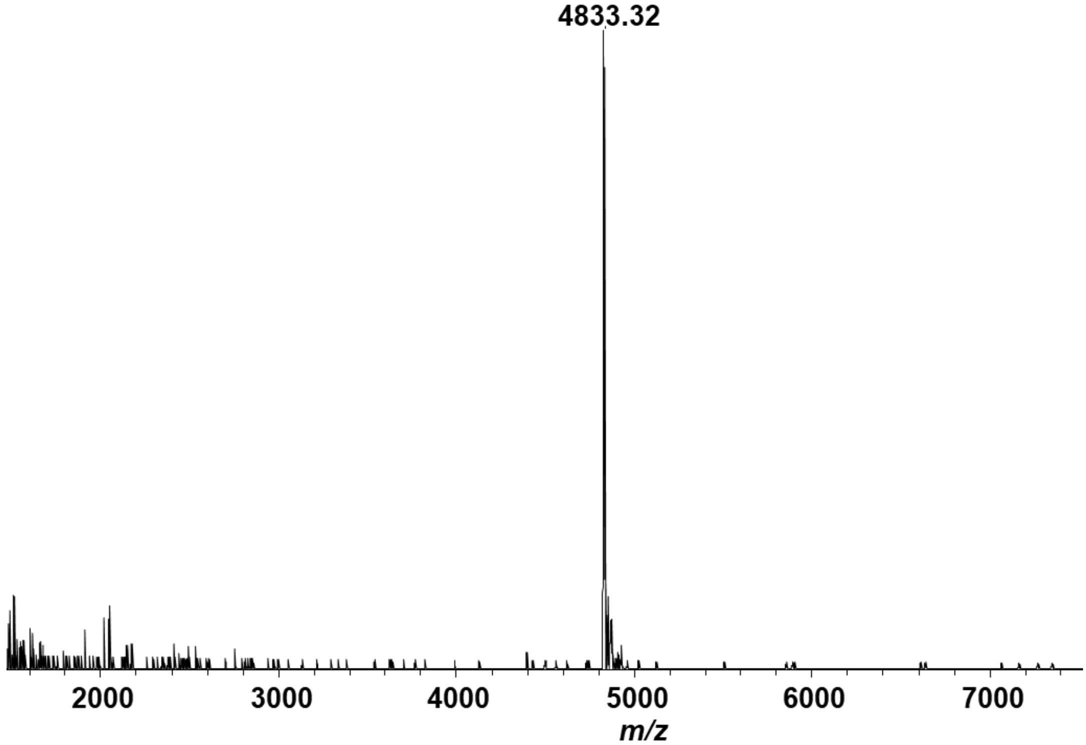


**Figure S18:** IEX-HPLC and MALDI-TOF MS analysis of purified hairpin **H5**

*IEX-HPLC analysis conditions: DNAPac® PA100, 4X250 mm, elution with a 20 min linear gradient of 5 to 40% of B in eluent A. Column temperature 30°C. Flow rate 1.0 mL.min$^{-1}$. λ 260 nm.*
*Eluant A : 25 mM Tris HCl, 5% ACN, pH 8*
*Eluant B : 25 mM Tris HCl, 400 mM $NaClO_4$, 5% ACN, pH 8*

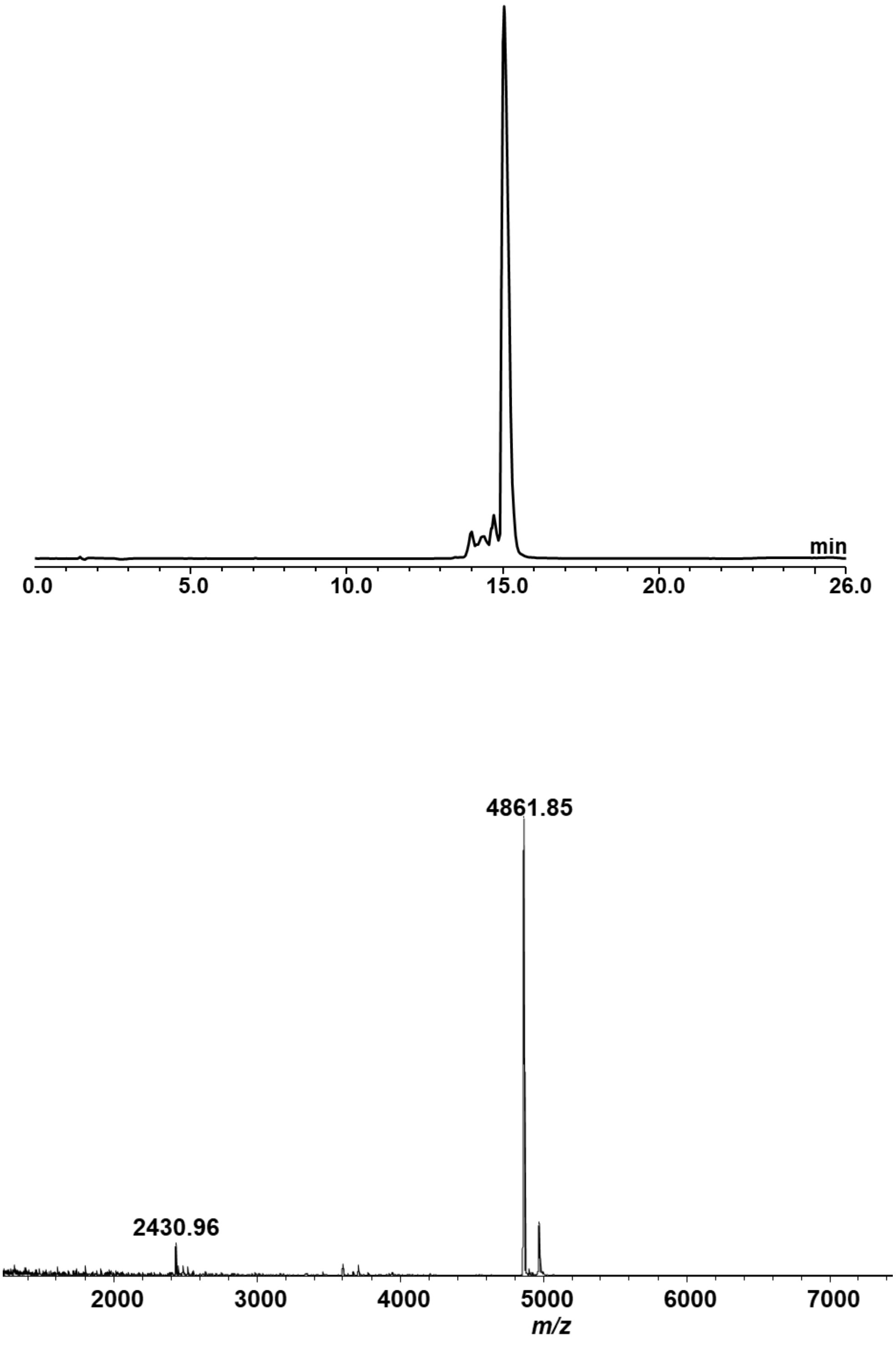


**Figure S19:** IEX-HPLC and MALDI-TOF MS analysis of purified hairpin **H6**

*IEX-HPLC analysis conditions: DNAPac® PA100, 4X250 mm, elution with a 20 min linear gradient of 5 to 50% of B in eluent A. Column temperature 75°C. Flow rate 1.0 mL.min$^{-1}$. λ 260 nm.*
*Eluant A : 25 mM Tris HCl, 5% ACN, pH 8*
*Eluant B : 25 mM Tris HCl, 400 mM $NaClO_4$, 5% ACN, pH 8*

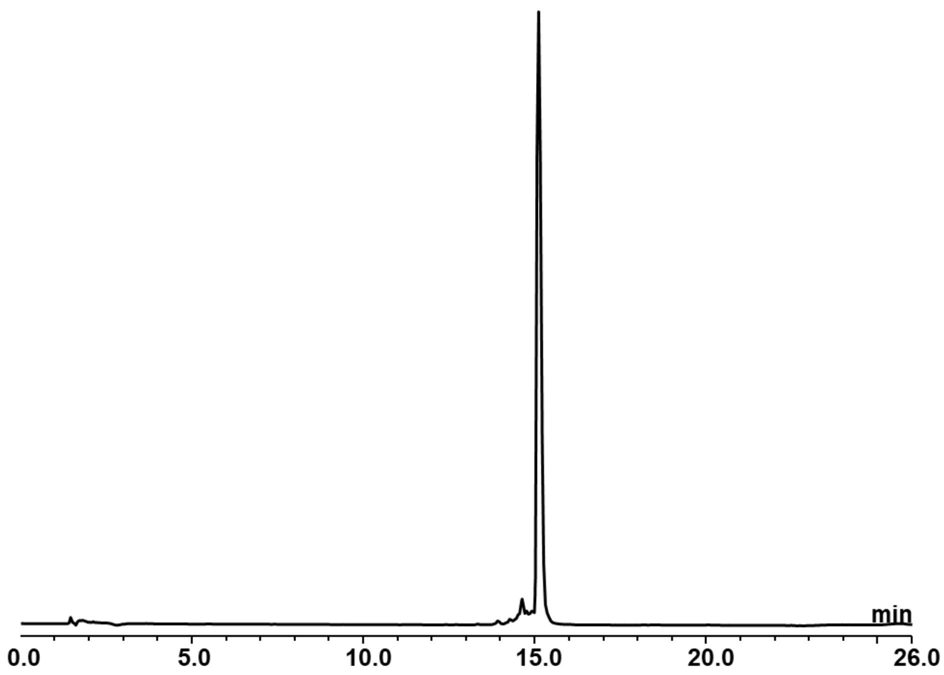


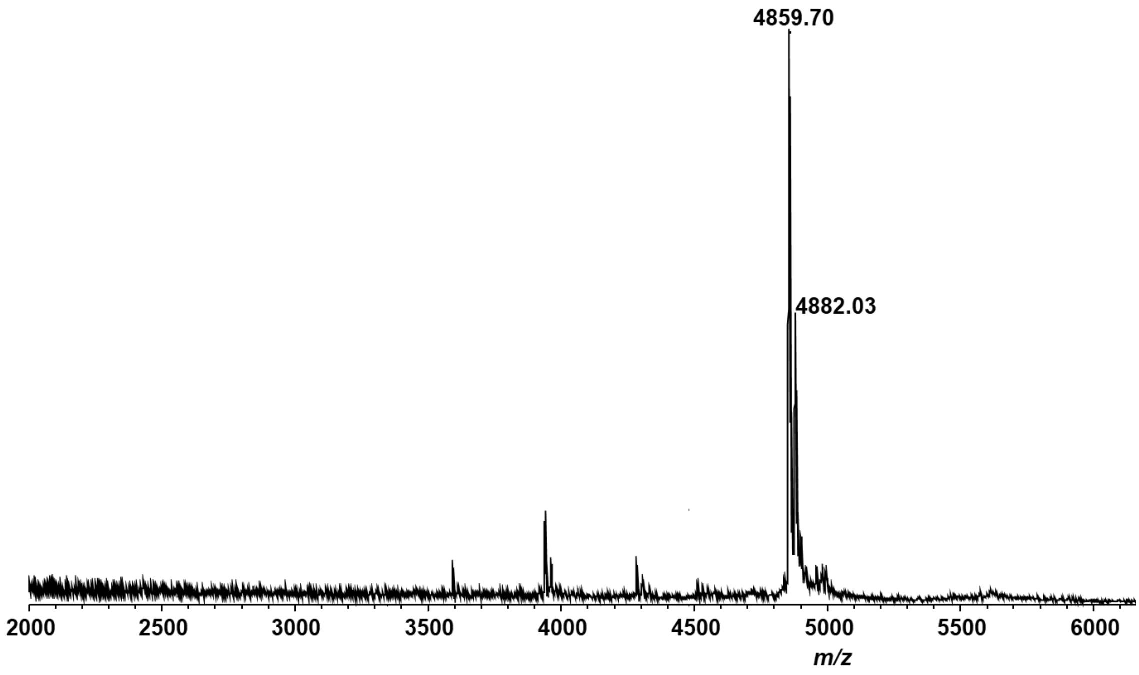


**Figure S20:** IEX-HPLC and MALDI-TOF MS analysis of purified hairpin **H7**

*IEX-HPLC analysis conditions: DNAPac® PA100, 4X250 mm, elution with a 20 min linear gradient of 5 to 50% of B in eluent A. Column temperature 75°C. Flow rate 1.0 mL.min$^{-1}$. λ 260 nm.*

*Eluant A : 25 mM Tris HCl, 5% ACN, pH 8*

*Eluant B : 25 mM Tris HCl, 400 mM $NaClO_4$, 5% ACN, pH 8*

## Circular Dichroism spectra

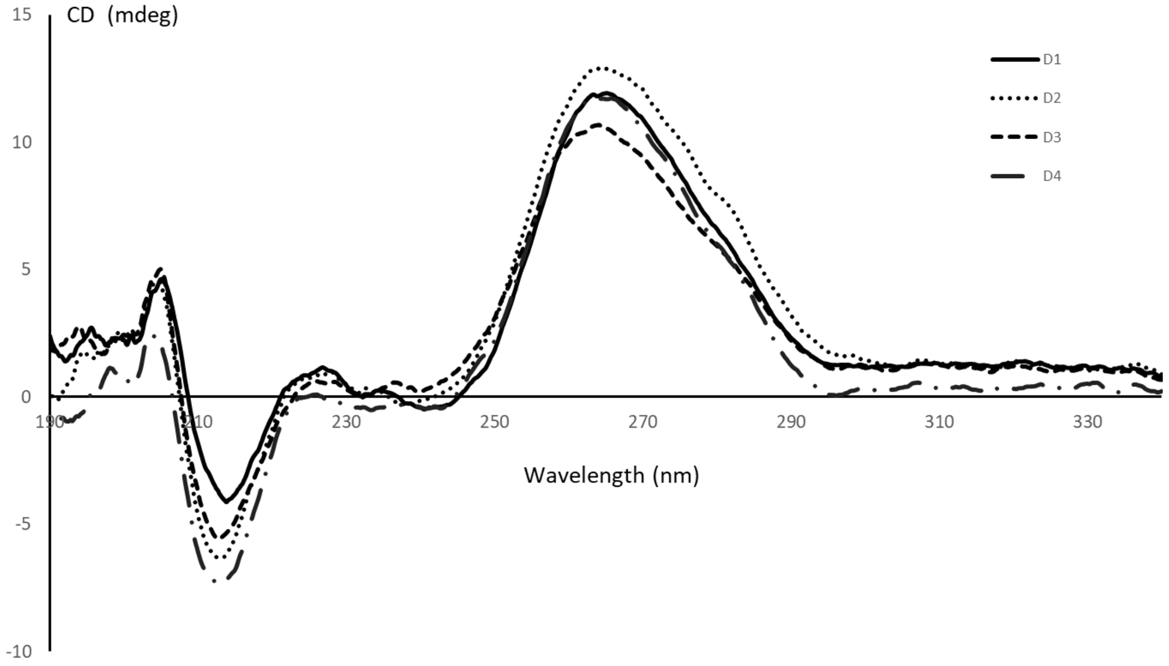


**Figure S21:** CD spectroscopy curves at 1°C of RNA/RNA duplexes **D1-D4** (Buffer: 10 mM sodium cacodylate, 100 mM NaCl, pH 7

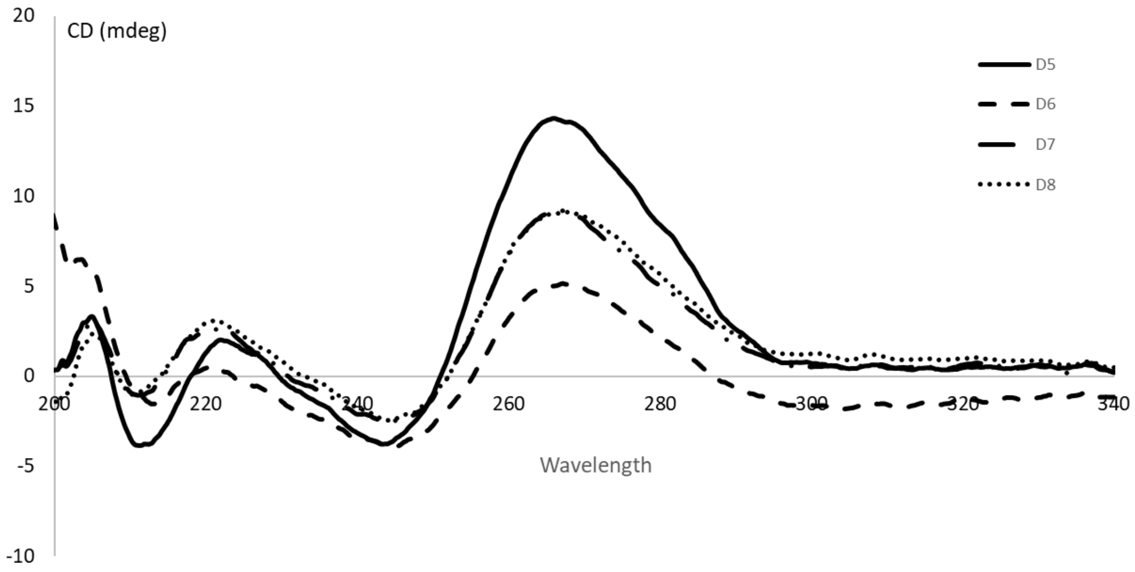


**Figure S22:** CD spectroscopy curves at 1°C of RNA/DNA duplexes **D5-D8** (Buffer: 10 mM sodium cacodylate, 100 mM NaCl, pH 7)

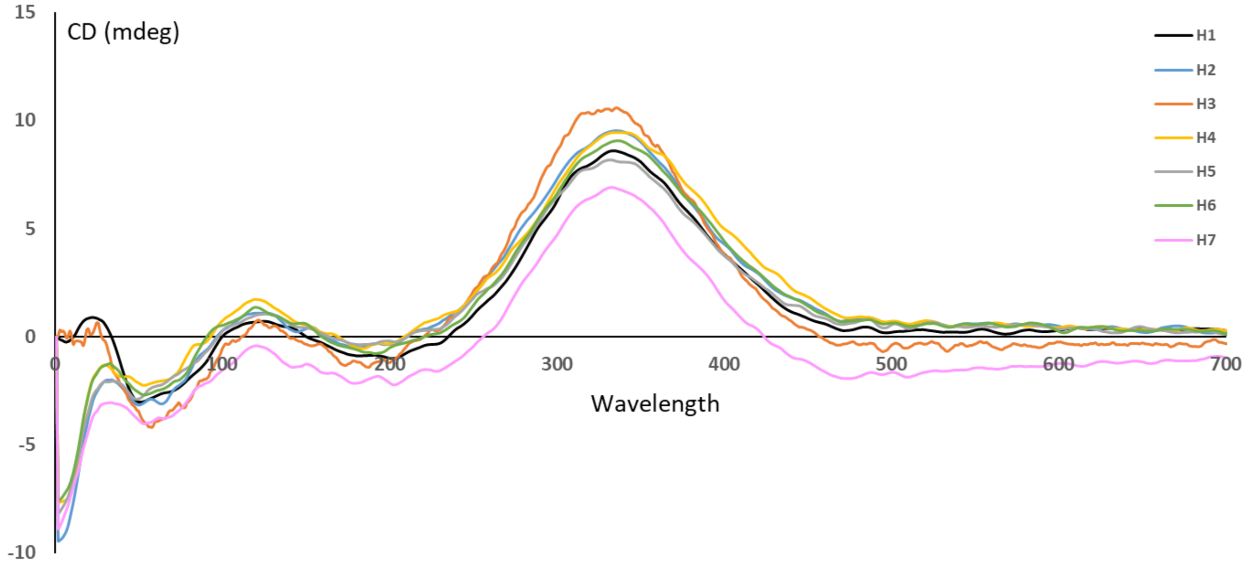


**Figure S23:** CD spectroscopy curves at 1°C of Hairpins **H1-H7** (Buffer: 10 mM sodium cacodylate, 100 mM NaCl, pH 7)

## Enzymatic stability of hairpin H3

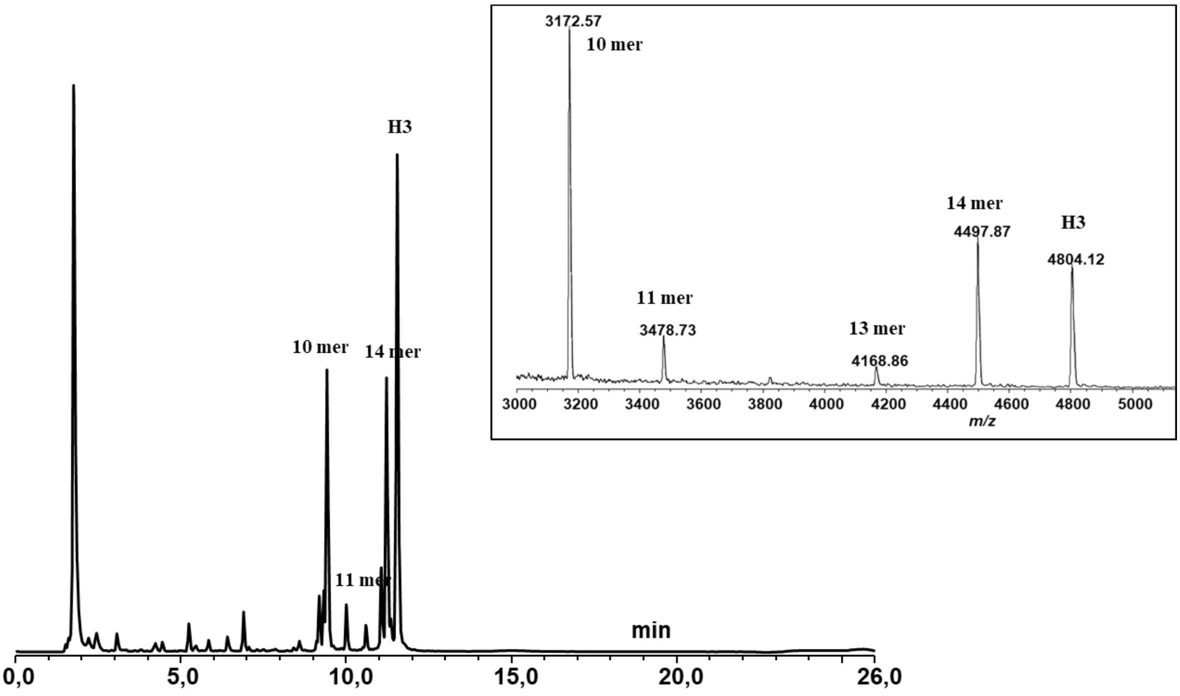


**Figure S24:** IEXP-HPLC analyses and MALDI-TOF MS spectra of hairpin **H3** incubated with SVPD at t = 120 min.

## Molecular docking simulation of dinucleotides UU

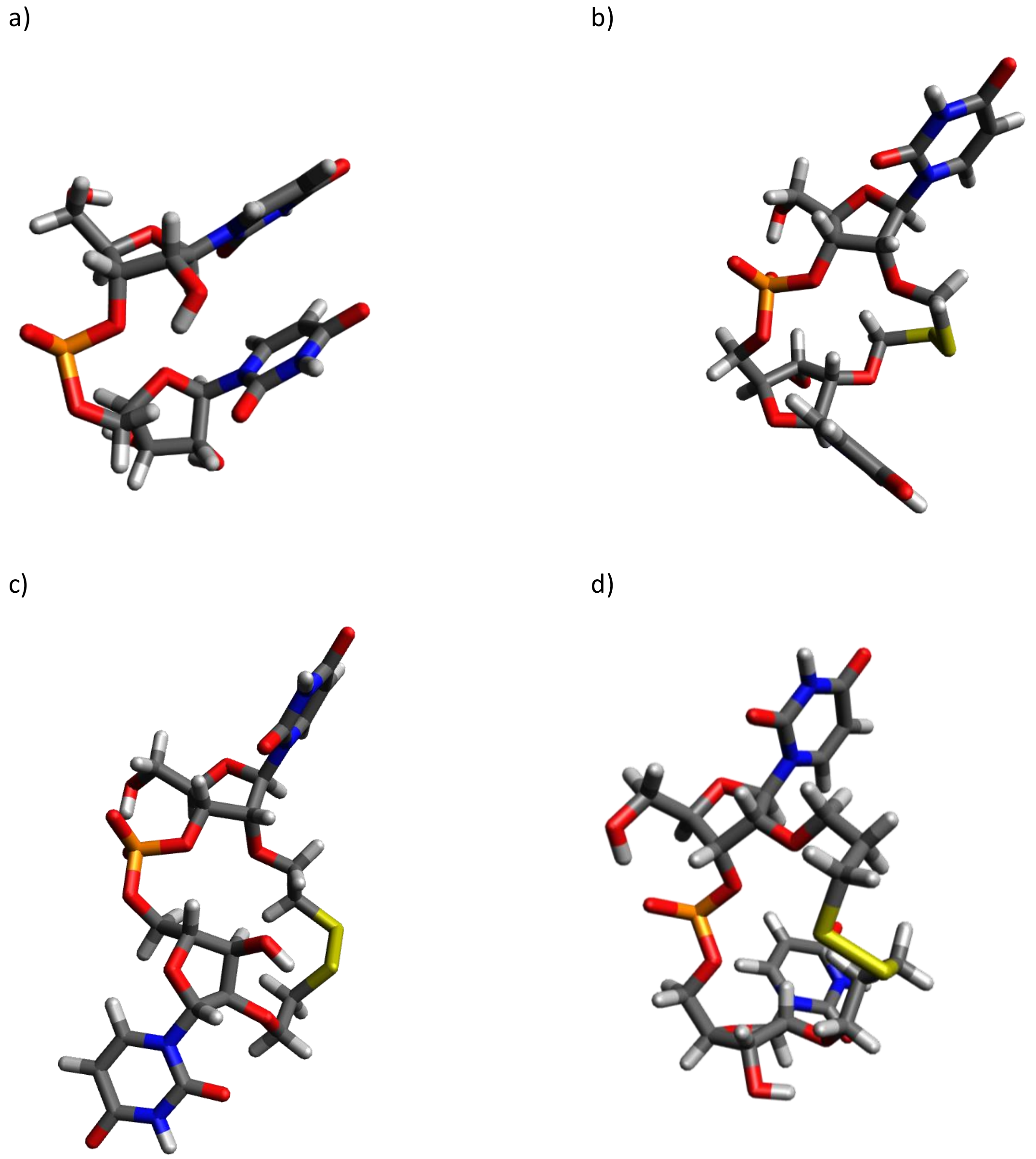


**Figure S25:** Molecular modeling of dinucleotides a) UU d b) 2',2' DME S-S bridge ; c) 2',2' DEE S-S bridge and d) 2',2' DPE S-S bridge.
All UU dinucleotides were drawn the 5'-end on top. Molecular docking simulations were performed using Avogadro an open-source molecular builder (Version1.2, http//Avogadro.cc)

| | UU | UU-2',2' DME S-S | UU-2',2' DEE S-S | UU-2',2' DPE S-S |
|---|---|---|---|---|
| d($C_{1'i}$-$C_{1'i=1}$) (Å) | 4.8 | 7.4 | 8.3 | 7.6 |
| $O_{3'}$-P-$O_5$-$C_{5'}$(deg) | -173 | -154 | -42 | 166 |
| $C_{3'}$-$O_{3'}$-P-$O_5$ (deg) | 51 | 178 | -175 | -141 |

**Table S2-:** Torsion angles α and ζ. and $C_{1'}$-$C_{1'i+}$distance of dinucleotides UU determined using Avogadro an open-source molecular builder (Version1.2, http//Avogadro.cc)
